# Ultra‐Broadband Optical Characterization of TiO_2_ Nanotube Arrays Revealing Annealing‐Tunable THz Absorption

**DOI:** 10.1002/nap2.70231

**Published:** 2026-07-29

**Authors:** Anjali Chaudhary, Morteza Afshar, Emil J. Y. Magaway, Sunil Kumar, Ravendra Kumar Varshney, Jiří Čapek, Ivan Richter, Štěpán Kment, Miguel Navarro‐Cía, Luca Mascaretti

**Affiliations:** ^1^ Department of Physics Indian Institute of Technology Delhi New Delhi India; ^2^ School of Physics and Astronomy University of Birmingham Birmingham UK; ^3^ Czech Advanced Technology and Research Institute Regional Centre of Advanced Technologies and Materials Palacký University Olomouc Olomouc Czech Republic; ^4^ Department of Solid State Engineering Faculty of Nuclear Sciences and Physical Engineering Czech Technical University in Prague Prague Czech Republic; ^5^ Department of Laser Physics and Photonics Faculty of Nuclear Sciences and Physical Engineering Czech Technical University in Prague Prague Czech Republic; ^6^ CEET Nanotechnology Centre VŠB‐Technical University of Ostrava Ostrava‐Poruba Czech Republic; ^7^ Department of Electronic, Electrical and Systems Engineering University of Birmingham Birmingham UK

**Keywords:** broadband absorbers, spaced titanium dioxide nanotubes, tunable THz absorption, ultrathin absorbers

## Abstract

Conventional terahertz (THz) absorbers often suffer from narrow bandwidths, bulky designs, and complex, multi‐step fabrication processes. By exploiting the extremely high phonon‐induced permittivity of titanium dioxide (TiO_2_) at THz frequencies, we report broadband absorbers based on spaced TiO_2_ nanotubes (NTs) fabricated by electrochemical anodization and thermal annealing featuring ∼ 80% absorptance from ∼10 THz up to 300 THz. Furthermore, the structures displayed a broad (∼1 THz wide) and tunable absorption peak in the 1.4–3.6 THz range by simply controlling the annealing time. To map the millimeter‐wave‐to‐ultraviolet spectrum and to uncover the dielectric response of the NTs, a multi‐instrument spectroscopy characterization strategy was combined with full‐wave electromagnetic simulations. The tunable and broadband absorption of spaced TiO_2_ NTs together with their high uniformity and scalable fabrication process open new opportunities for multipurpose low‐cost absorbers and compact room‐temperature THz devices.

## Graphical abstract

Lithography‐free spaced TiO_2_ NT arrays unlock tunable broadband absorption from THz to UV. By harnessing annealing‐driven phase transitions, strong phonon–polariton resonances emerge in the 1.4–3.6 THz range, whereas high absorptance persists beyond 10 THz. This scalable oxide platform paves the way for compact, multifunctional devices in THz imaging, spectroscopy, and optoelectronic energy conversion.

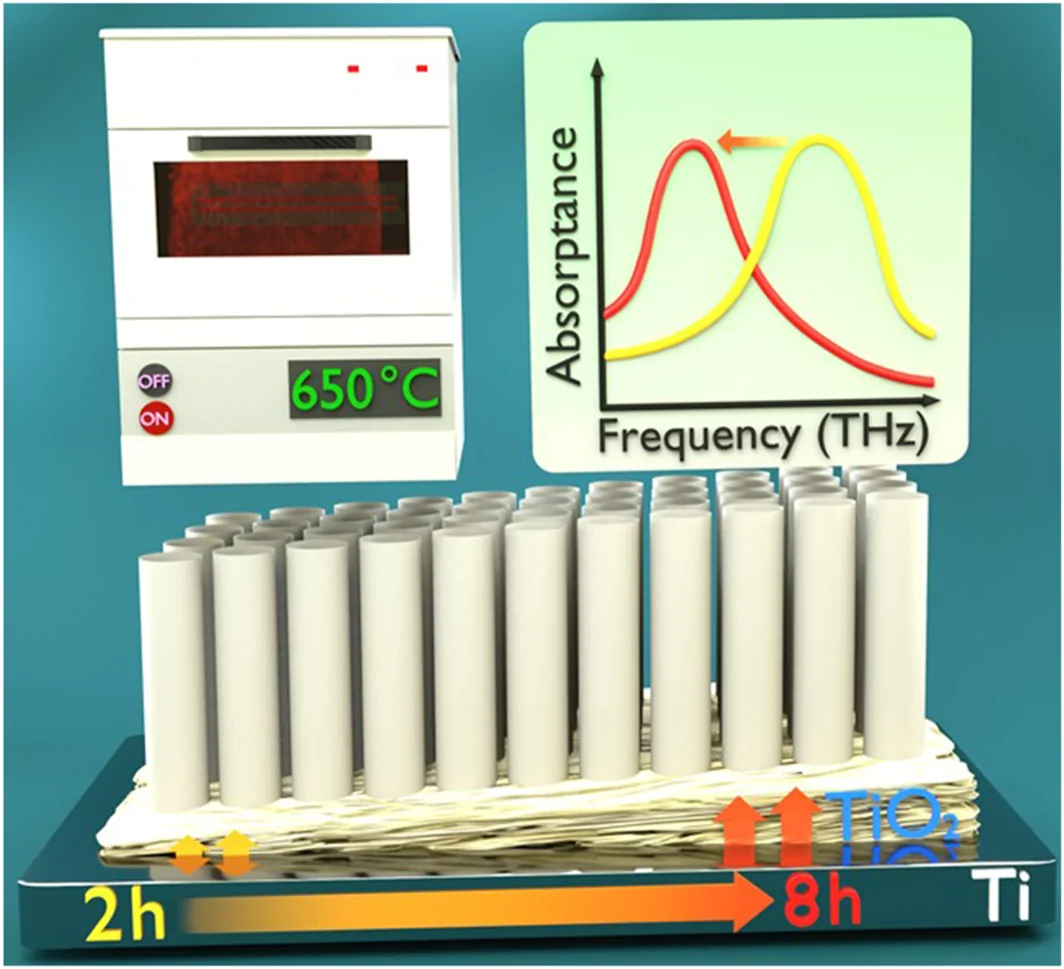

## 1 Introduction

The terahertz (THz) frequency region bridging the microwave and infrared domains has become a rapidly advancing frontier in spectroscopy, imaging, and communication [1, 2, 3]. At these long wavelengths, achieving strong light–matter interaction within sub‐micron‐thick materials remains challenging because most conventional dielectrics exhibit low permittivity and medium absorption as well as because of the length‐scale mismatch between the THz wavelength (sub‐mm to micron) and the physical interaction length (sub‐micron). Consequently, most THz absorbers developed so far that are not electrically thick rely on metallic metasurfaces, where resonant inductive–capacitive (*LC*) elements coupled to a metallic ground plane produce near‐perfect absorption through impedance matching to free space [4, 5].

Previously reported metasurface absorbers suffer from bandwidth constraints, as illustrated by the work of Kuznetsov et al., who demonstrated a polarization‐selective metasurface absorber for pyroelectric millimeter‐wave detection operating around 140 GHz [6]. This metasurface absorber had a deep subwavelength thickness (∼15 μm), but the absorption bandwidth remained limited to ∼5% due to the resonant *LC* nature of the metallic metasurface design. To overcome these limitations, alternative absorber concepts based on intrinsically lossy materials have been explored. For instance, flexible vertically aligned carbon‐nanotube (VACNT) arrays have been employed for real‐time THz beam profiling and imaging, achieving average absorptance above 96% in the 0.1–2.5 THz range [7]. However, their fabrication typically requires complex chemical‐vapor‐deposition and delicate transfer processes. Another strategy relies on biomimetic textured surfaces that suppress reflection; for example, a “moth‐eye” absorber consisting of a silicon microscale texture coated with pyrolytic carbon enables absorption exceeding 98% over an extremely broad range from 1 to 1200 THz [8]. Nevertheless, such structures generally require lithographic patterning and carbon deposition, increasing fabrication complexity and cost.

The limitations of metallic metasurfaces and carbon‐based architectures motivated the search for alternative materials and simpler designs for THz absorbers. For example, titanium dioxide (TiO_2_) has been largely investigated as a prototypical photocatalyst material [9, 10] and high‐index dielectric for antireflection coatings [11]. Moreover, both its common polymorphs, that is, anatase and rutile [12], offer a promising combination of intrinsic properties including high optical refractive index [13], strong lattice polarizability [14], and wide bandgap [15] that could be exploited for THz absorbers. In the THz regime, TiO_2_ exhibits strong dielectric losses associated with infrared‐active optical phonons. These lattice vibrations produce phonon–polariton excitations that lead to a large dielectric permittivity ($\varepsilon_{r}$ > 200) and efficient electromagnetic energy dissipation even in deeply subwavelength structures [16, 17, 18]. As a result, ultrathin TiO_2_ layers can act as effective THz absorbers by converting incident electromagnetic energy into heat through phonon‐mediated processes. Unlike for metallic metasurface absorbers, this mechanism relies on the intrinsic phononic response of the oxide rather than resonant *LC* structures, thus making TiO_2_ an attractive candidate for compact THz absorbers.

A further advantage of TiO_2_ is the possibility of realizing self‐organized nanostructured arrays, which may help engineer the absorptance using a simple and scalable electrochemical anodization method [19, 20]. The so‐called spaced TiO_2_ nanotubes (NTs) have been recently achieved by controlling the anodization parameters, such as applied voltage and water content in the electrolyte [21, 22]. Spaced NTs feature a higher surface area and inter‐tube periodicity than their close‐packed counterparts, which introduce multiple scattering paths and microcavity resonances that broaden the absorption spectrum at visible frequencies. These features have been exploited for applications in photoelectrochemistry [23] and surface‐enhanced Raman scattering [24]. However, the properties of spaced TiO_2_ NTs in the medium and far infrared regions remain unexplored, where multi‐resonant absorption mechanisms may arise from the interplay between the intrinsic phonon response of TiO_2_ and NT architecture. This could potentially lead to applications in THz devices.

In this work, we report the absorption properties of spaced TiO_2_ NT arrays over an ultra‐broadband wavelength interval, that is, from hundreds of nm (UV) to a few mm (THz). By engineering the morphological and structural properties via annealing treatments, we demonstrate a systematic tuning of a broad absorption peak in the range 1.4–3.6 THz both in terms of intensity and spectral position. These experimental findings were interpreted by numerical simulations in terms of the intrinsic electric polarizability of TiO_2_, which is related to phonon–polariton modes, and cavity confinement, which was attributed to the NT morphology. Such a combination of effects enabled effective impedance matching and electromagnetic energy confinement without ohmic losses. Consequently, these structures bridge the gap between conventional metamaterial absorbers and naturally resonant oxides, providing a scalable and cost‐effective approach to THz functional materials.

## 2 Results and Discussion

The realization of the multi‐frequency absorber was initially achieved through the anodization of Ti foil in a two‐electrode electrochemical cell by applying a 60 V potential between the electrodes for 3 h at 40°C. The as‐anodized spaced TiO_2_ NTs exhibited well‐aligned and distinct tubular morphology, with a mean tube length of ∼ 5 μm, center‐to‐center spacing of ∼ 480 nm, and outer and inner diameters of ∼ 275 and ∼ 245 nm, respectively. The initially amorphous NTs were crystallized by thermal treatment in air at 650°C, in agreement with previous work [19, 20]. Upon annealing, the formation of a compact oxide layer was observed beneath the NT arrays because of oxidation of the Ti substrate at the NT/Ti interface (Figure 1). The thickness of this oxide layer increased from ∼ 1.5 to ∼ 2 and ∼ 3 μm as the annealing time was increased from 2 to 4 and 8 h, respectively, although some nonuniformities were noticeable (Supporting Information S1 Figure S1). Meanwhile, the mean NTs' length remained unchanged at ∼ 3.8 μm for all annealing durations (Figure 1), as well as their overall morphology (Supporting Information S1 Figure S2).

**FIGURE 1:**
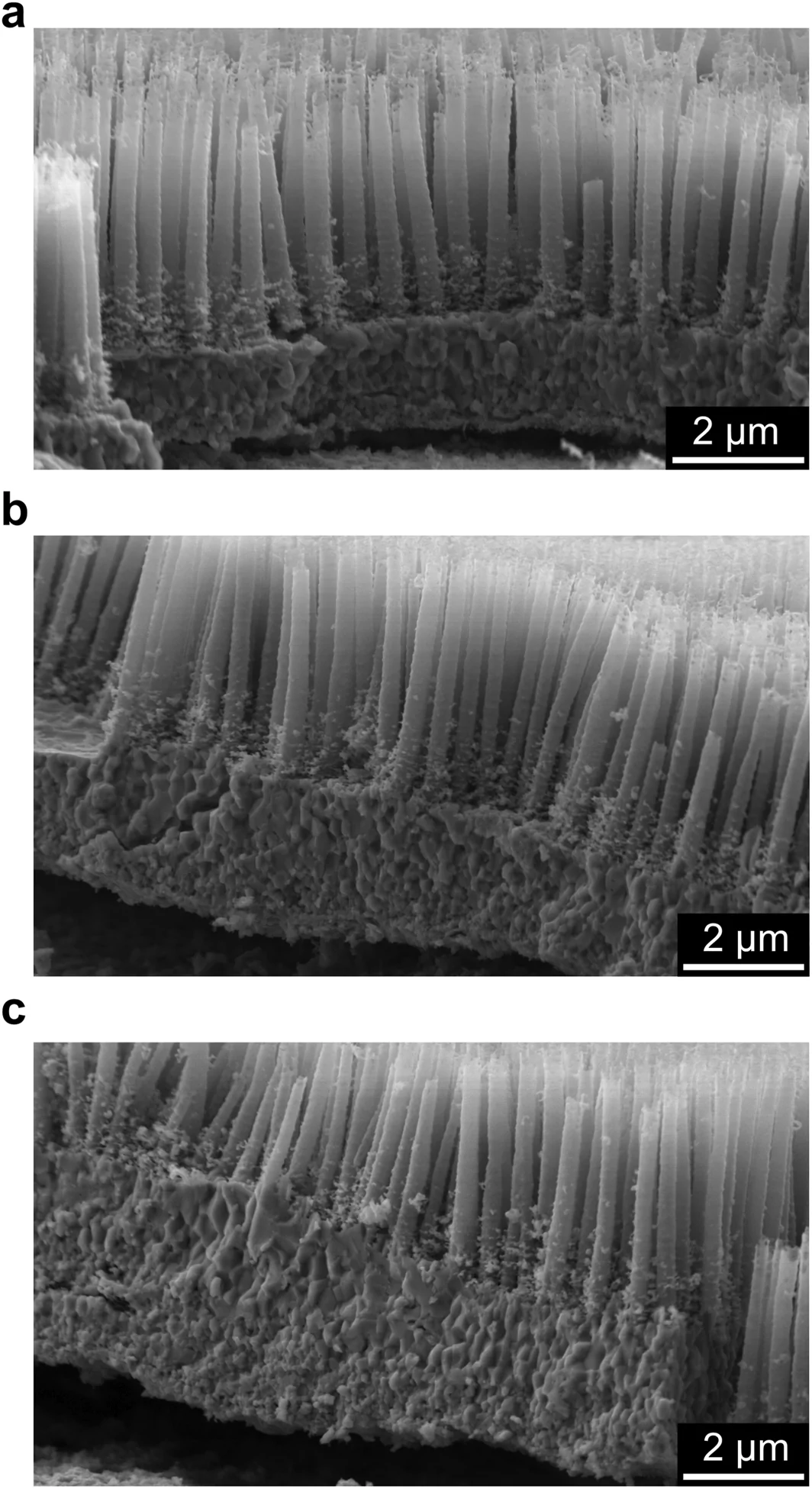
Morphology of spaced TiO_2_ NTs annealed in air at 650°C for (a) 2 h, (b) 4 h, and (c) 8 h.

The structural characterization of the annealed samples was addressed by XRD measurements in Bragg‐Brentano configuration to probe the whole thickness of the NTs (Figure 2). The diffraction patterns revealed the presence of the two main crystalline phases of TiO_2_, that is, anatase and rutile, where the latter was the prevalent phase (see Supporting Information S1 Table S1 for the assignment of all diffraction peaks). Previous studies have shown that anodic amorphous NTs can be crystallized into anatase upon air annealing at temperatures higher than 300°C, whereas the rutile phase appears after 500°C [19]. As a result, mixed‐phase TiO_2_ NTs can be obtained by thermal treatments at temperatures higher than 500°C, with a relative fraction of rutile increasing with the annealing temperature and duration [19]. The growth of a compact rutile layer underneath the NTs at annealing temperatures > 550°C is also consistent with previous work [19, 25]. In the case of spaced TiO_2_ NTs annealed at 650°C, the relative content of anatase slightly decreased with the annealing time (from top to bottom of Figure 2). Indeed, the relative anatase fraction was estimated by the empirical Spurr‐Myers formula [26],

(1) $$x_{a}=\frac{1}{1+1.26\cdot I_{r}/I_{a}}$$

where *I*_*r*_ is the intensity of the (110) rutile peak (main peak at ∼ 32°), and *I*_*a*_ is the intensity of the (101) anatase peak (main peak at ∼ 29.5°). The fraction of anatase decreased from ∼ 29% (2 h) to ∼ 17% (8 h), and the same results were obtained by Rietveld refinement of diffraction patterns. This may be attributed to the onset of the anatase‐rutile phase transition at the bottom interface of the NTs with the Ti substrate, where a rutile layer forms and acts as an initiation layer for that phase transition [25, 27, 28]. The average crystallite size for the rutile phase estimated by the Rietveld refinement method increased with the annealing time from ∼ 230 to ∼ 340 nm, whereas that of the anatase phase remained approximately constant at ∼ 110 nm (Supporting Information S1 Figure S3). At the same time, the compact layer below the NTs (Figure 1) increased in thickness with the annealing time and consisted of pure rutile. Therefore, the increase of the relative amount of rutile with the annealing time may be explained by the anatase‐rutile phase transition in the NTs as well as by the increasing thickness of the compact bottom layer beneath. The emergence of the compact rutile layer and its growth with the annealing time at 650°C produced an impact on the optical behavior of the samples, as shown below.

**FIGURE 2:**
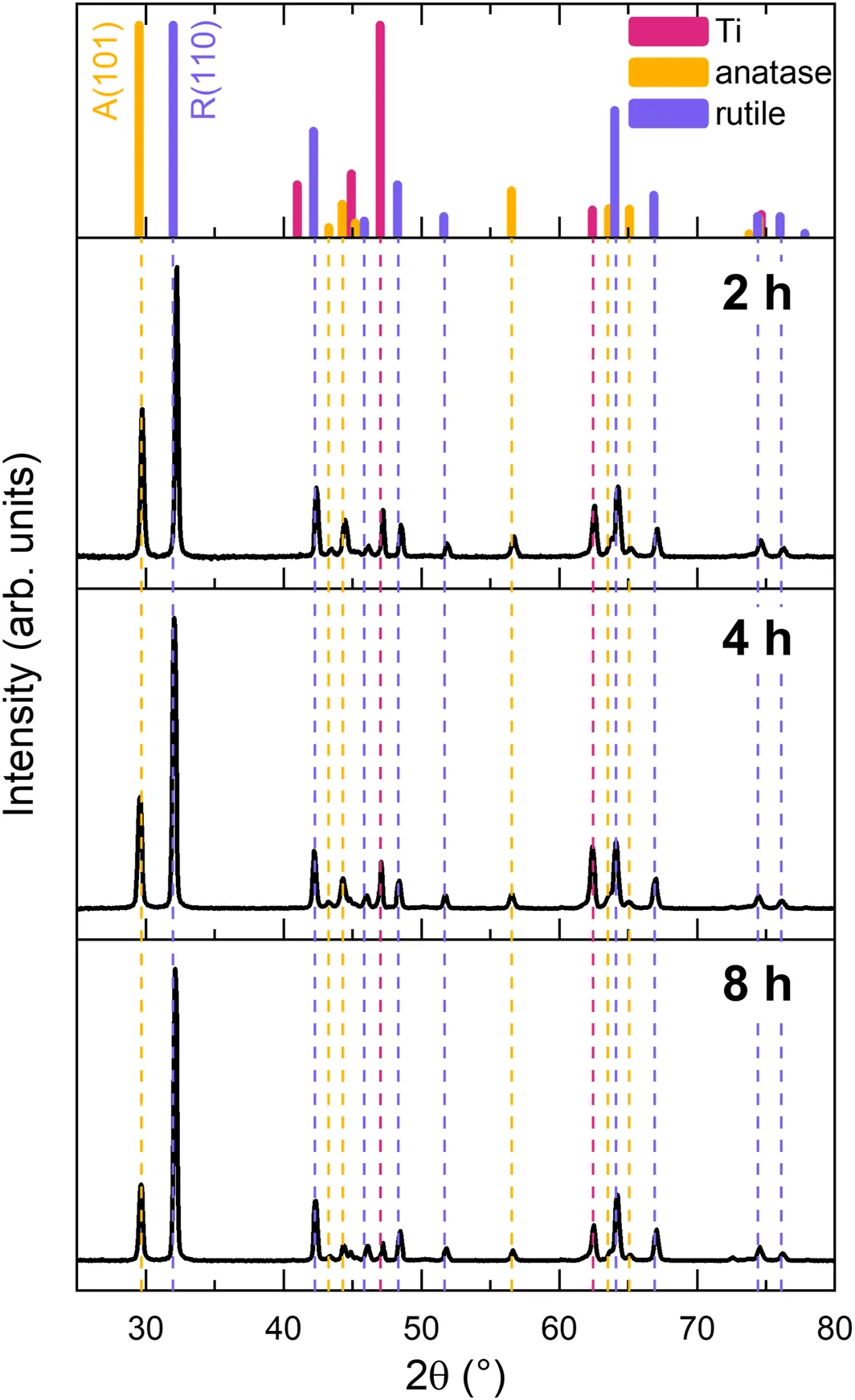
X‐ray diffraction patterns of spaced TiO_2_ NTs annealed in air at 650°C for 2 h (top), 4 h (middle), and 8 h (bottom) compared to the reference patterns (top panel) of hexagonal Ti (PDF card #00‐044‐1294), anatase (PDF card #01‐89‐4921), and rutile (PDF card #00‐021‐1276).

An ultra‐broadband optical characterization of absorption phenomena in spaced TiO_2_ NTs was addressed by employing a combination of experimental setups, which included a spectrophotometer, an FTIR spectrometer, a continuous wave (CW) photomixer, and a THz time domain spectroscopy (TDS) system (Figure 3, see Experimental methods for additional details and Supporting Information S1 Figure S4 for the raw reflectance curves of the 2 h–annealed sample). The spectrophotometer (Figure 3a) was employed to capture the absorptance in the UV–visible–NIR range, whereas the vacuum FTIR system (Figure 3b) allowed retrieving the same quantity over a region spanning from the mid‐ to the far‐infrared. The CW THz photomixing setup (Figure 3c) and the THz‐TDS system (Figure 3d) were employed to measure the THz absorption by detecting the specular reflection of an incident focused beam onto the sample. These complementary techniques collectively enabled spectral coverage from 0.1 THz to 1000 THz, therefore providing information on the optical absorption features of spaced TiO_2_ nanotubes over an unprecedentedly wide frequency window.

**FIGURE 3:**
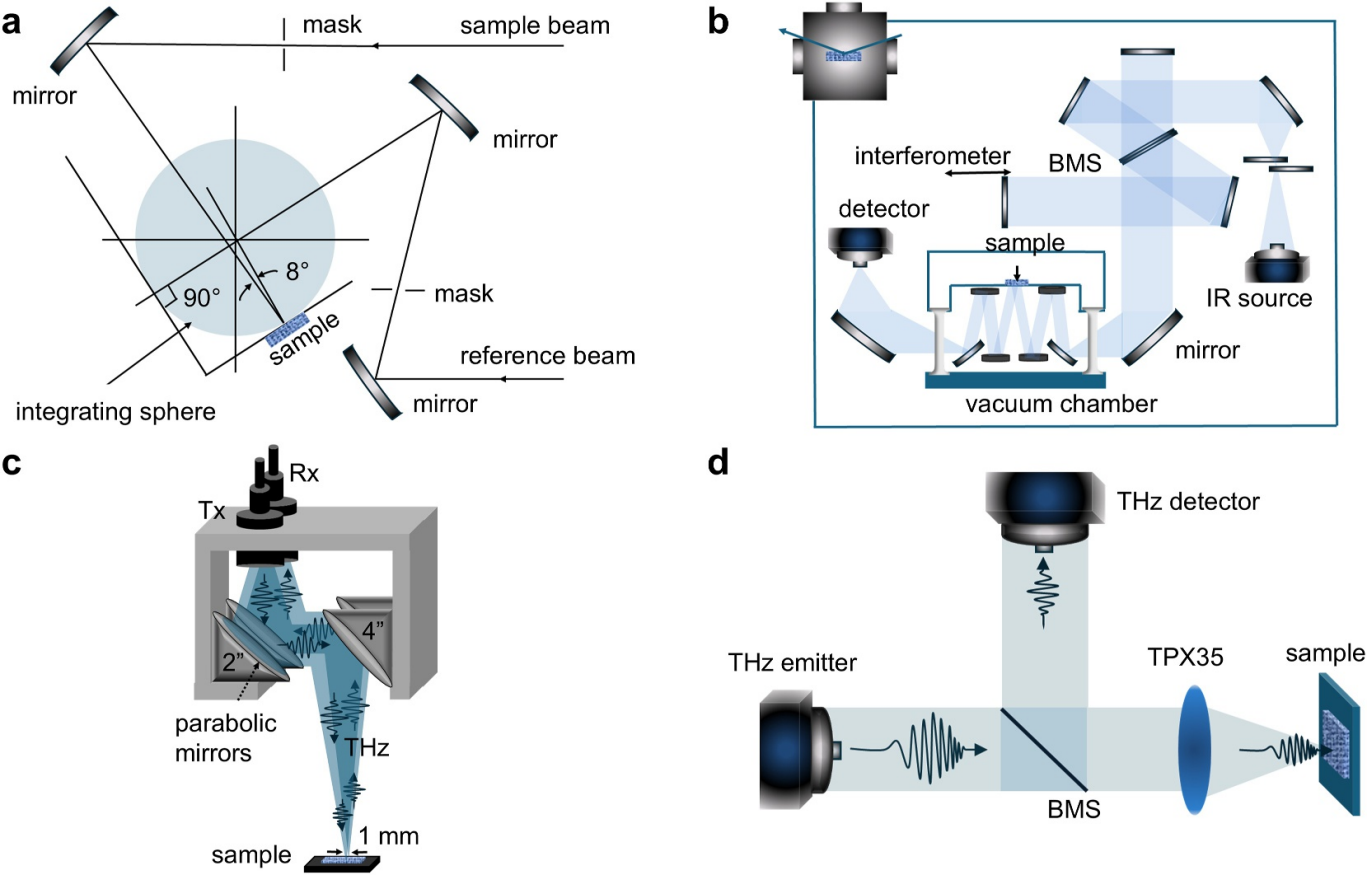
Schematic of the instrumentation used for the optical characterization of the spaced TiO_2_ NTs, (a) spectrophotometer, (b) vacuum FTIR spectrometer, (c) CW photomixer, and (d) TDS.

The optical properties of as‐anodized and annealed spaced TiO_2_ NTs were measured by the combination of methods mentioned above to reveal the influence of thermal annealing on their response over an ultra‐broad frequency range (Figure 4), with a specific interest in their resonant response in the THz regime. The as‐anodized sample (Figure 4a) exhibited low absorption in the THz range, which can be interpreted in terms of weak lattice polarization and limited optical phonon activity of the amorphous structure. On the contrary, all annealed films exhibited a well‐defined absorption peak in the THz region. Specifically, for the 2 h–annealed film, a strong resonance was observed at 2.62 THz with ∼75% absorptance (Figure 4b). For the 4 h–annealed sample, the peak redshifted to 2.46 THz with 77% absorptance (Figure 4c), whereas the 8 h annealing produced a further redshift to 1.67 THz as well as an increase of absorptance up to 87% (Figure 4d). In other words, the increase of annealing time produced a systematic redshift and intensity enhancement of the absorption peak in the THz region. In addition, the bandgap transitions of anatase (3.2 eV) [29] and rutile (3.0 eV) [15] became well‐distinguishable in the case of the 8 h–annealed sample (Figure 4d), thus confirming the progression of the phase transition to rutile revealed by XRD analysis (Figure 2).

**FIGURE 4:**
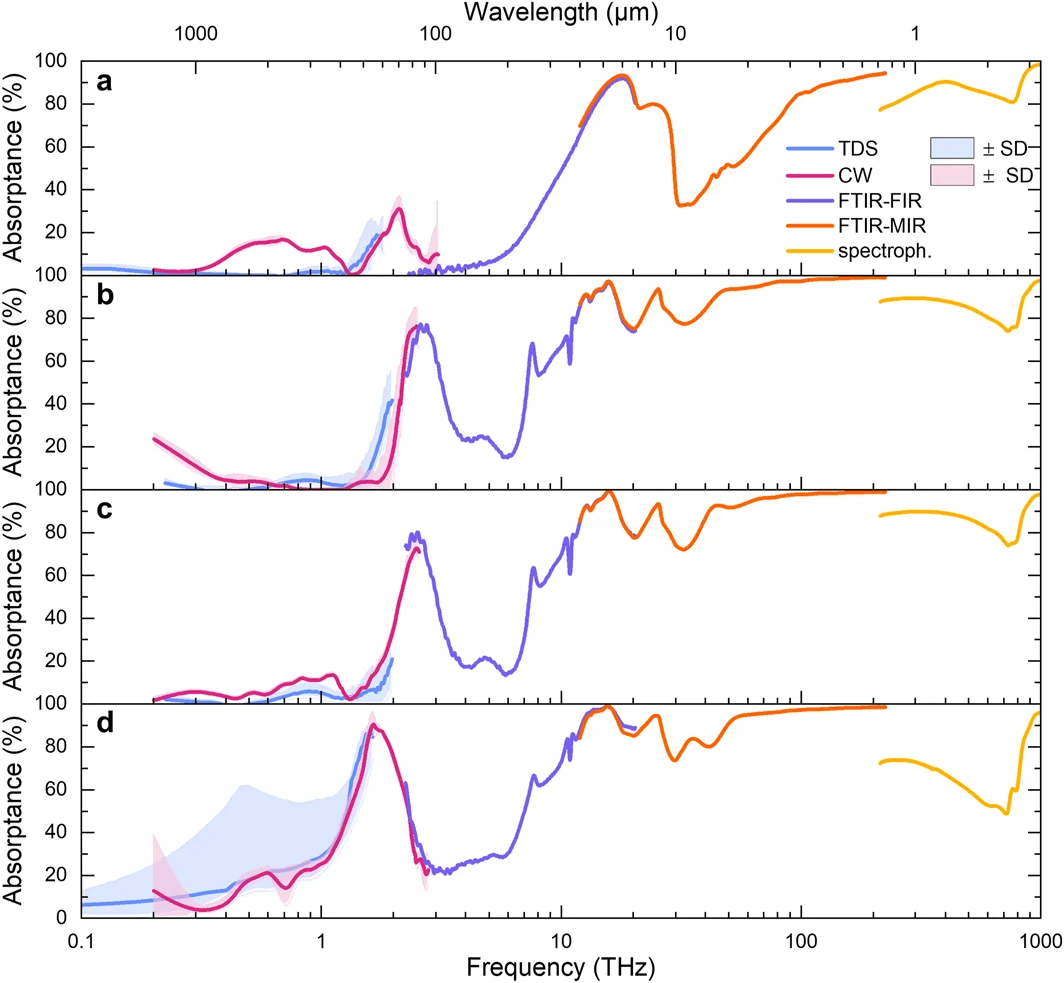
Broadband optical absorption of as‐anodized (a) and annealed (b–d) spaced TiO_2_ NTs for different annealing durations, 2 h (b), 4 h (c), and 8 h (d). The shaded regions in the TDS and CW datasets represent standard deviations and confirm the repeatability of the experimental measurements.

Furthermore, all annealed samples exhibited a high absorption in the whole medium‐IR range with some maxima in the 15–50 THz region and with a rather featureless quasi‐linear trend in the 50–200 THz region. This consists of a pronounced enhancement compared to the as‐anodized NTs, which exhibited ∼40% absorption in the 10–30 THz range, which can be explained by the change in the dielectric response of the material [15, 16]. Indeed, unlike amorphous TiO_2_, the crystalline phases support well‐defined infrared‐active optical phonon modes associated with Ti–O lattice vibrations, resulting in stronger coupling with incident mid‐infrared radiation and enhanced dielectric damping [16, 27, 30, 31]. Consequently, electromagnetic energy is dissipated more efficiently within the oxide layer, leading to increased absorptance. As annealing progresses, the increasing rutile fraction and improved crystallinity further strengthen these phonon‐mediated loss mechanisms. Furthermore, at frequencies above ∼10 THz, additional infrared‐active vibrational modes and higher‐order phonon‐related absorption processes contribute to increased dielectric losses, sustaining the high absorptance observed across the mid‐infrared spectral region [18, 32, 33].

The well‐defined absorption peak in the THz region observed for all annealed films may be interpreted in terms of phonon polariton modes. Rutile TiO_2_ possesses higher static dielectric permittivity and stronger IR‐active transverse optical (TO) phonons than anatase due to increased ionic polarizability and long‐range dipolar coupling [30, 34]. When polar crystals are exposed to THz radiation, the incident photons can couple to collective lattice vibrations mediated by longitudinal–transverse phonon splitting [30, 32, 34, 35], which gives rise to surface or volume phonon–polariton modes. Experimentally, this corresponds to the absorption peak arising between 1.6 and 2.6 THz in Figure 4. As the rutile content increased, these resonances became more pronounced due to enhanced oscillator strength and narrowed phonon linewidths. In other words, the peak intensity increased thanks to improved crystallinity and reduced phonon damping. Moreover, the observed redshift can be attributed to phonon softening, that is, a reduction in TO phonon frequency caused by internal strain relaxation and modified Born effective charges during the phase transition [16, 18, 30, 32, 34, 35]. Therefore, a simple increase in thermal annealing time promoted stronger light–matter interactions at the phonon–polariton resonance, thus allowing a precise spectral control of the absorption peak in the THz region in terms of both frequency and intensity.

The interpretation of absorption features in the THz region was further addressed by reproducing the experimental spectra with numerical simulations (Figure 5). The model was built as a square array of NTs with the same geometrical parameters for all samples and with a compact layer below the NT array with a variable thickness corresponding to the rutile layer, where all dimensions of the model parameters were consistent with the fabricated samples (Figure 1, more details in the Experimental Methods section). The dielectric function of TiO_2_ for both the NTs and the compact rutile layer was represented with the phenomenological Lorentz model (Equation 2), which represents phonon–polariton interactions in this frequency region [17]. The model parameters for all annealed spaced TiO_2_ NTs (Table 1) were obtained starting from literature values for rutile TiO_2_ [17] and by fitting the Lorentz model to the experimentally observed THz absorption peak. The relatively small variation of the fitted Lorentz oscillator parameters is physically reasonable because all annealed samples consist predominantly of the rutile TiO_2_ phase. Consequently, the intrinsic dielectric response remains largely unchanged, whereas the evolution of the THz absorption spectra is mainly governed by annealing‐induced morphological modifications. The simulated and experimental absorptance spectra for all annealed films are shown in Figure 5a. The simulated peaks aligned closely with the measured resonance frequencies (2.62, 2.46, and 1.67 THz for 2, 4, and 8 h–annealed samples, respectively), demonstrating that the modeling based on a periodic array of NTs and a simple Lorentz oscillator for the dielectric function was sufficient to capture the absorption physics. The model assumes an ideal periodic nanotube array with a homogeneous dielectric response; explicitly incorporating all morphological and structural nonidealities of the fabricated samples would be computationally impractical. Discrepancies in linewidth and spectral shape between simulations and experiments can be attributed to morphological inhomogeneities, including nonideal NT periodicity, tube distortions or tilting, substrate roughness, and variations in the compact rutile layer thickness (Figure 1, Supporting Information S1 Figures S1 and S2), as well as to structural inhomogeneities, that is, presence of both anatase and rutile phases (Figure 2). All these effects may likely result in a broader experimental absorption peak in the THz region compared to the simulations and may also mask higher‐order resonances predicted for thicker oxide layers. Additional simulations were thus performed varying the dimensional parameters of the model. The THz absorption redshifted by increasing the compact TiO_2_ layer thickness below the NTs (Supporting Information S1 Figure S5), so that the experimental peak broadening was used to estimate a ± 0.4 μm thickness deviation for the 2 h– and 4 h–annealed NTs and ± 0.8 μm for the 8 h–annealed NTs, respectively (Supporting Information S1 Figure S6), which is consistent with the thickness variations directly measured by SEM (Figure 1). On the other hand, the THz absorption peak was not affected by varying the NTs diameter (Supporting Information S1 Figure S7), height (Supporting Information S1 Figure S8) and pitch (Supporting Information S1 Figure S9), which may be ascribed to the deep subwavelength size of all these parameters (max. ∼ 4 μm) compared to the THz wavelength (∼150 μm at 2 THz). An additional experimental nonideality consists of the inherent presence of off‐normal components in the focused THz beam during measurements, which may contribute to minor spectral broadening and slight resonance shifts [36, 37]. This effect was investigated by computing the angular dependence of the THz absorption peak, but minor spectral shifts were found only at very high angles of incidence (Supporting Information S1 Figure S10). Therefore, this analysis showed that the experimentally observed broadening mainly emerged from a nonuniform growth of the compact rutile layer below the NTs during thermal treatments, which introduced a distribution of local resonance conditions across the sample surface.

**FIGURE 5:**
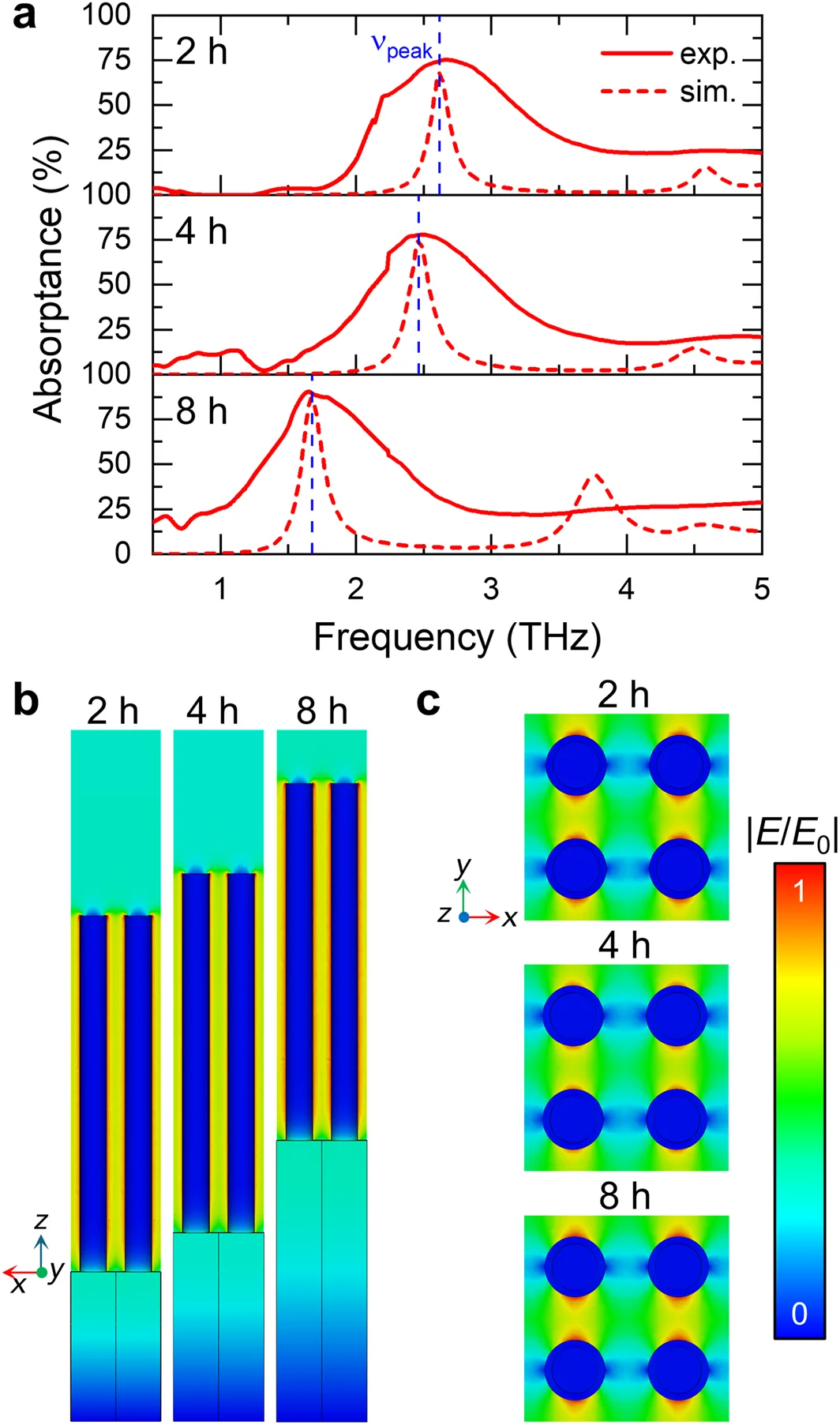
(a) Experimental (solid lines) and simulated (dashed lines) optical absorption spectra in the THz region for the spaced TiO_2_ NTs annealed for 2, 4, and 8 h. Dashed vertical lines mark the peak resonance frequency (ν_peak_). (b) Side view and (c) top view of simulated electric field maps for the annealed spaced TiO_2_ NTs at their respective ν_peak_ normalized by the maximum value of the electric field in the simulation domain (*E*_*0*_).

**TABLE 1:** Lorentz oscillator model parameters for TiO_2_ NTs at different annealing durations.

Annealing time	High‐frequency permittivity ($\varepsilon_{\infty }\;)$	Static permittivity ($\varepsilon_{s}$)	Resonance frequency $\omega_{0}$ (rad s^−1^)	Damping constant, δ ($s^{-1}$)
Rutile TiO_2_ [17]	6.85	128	$3.33\times 10^{13}$	$1\times 10^{12}$
2 h	6.20	240	$3.33\times 10^{13}$	$2\times 10^{12}$
4 h	6.20	180	$3.33\times 10^{13}$	$3\times 10^{12}$
8 h	6.20	200	$3.33\times 10^{13}$	$4\times 10^{12}$

Further insights into the THz absorption mechanism in annealed TiO_2_ NT arrays were addressed by computing the simulated electric field distributions at the respective peak absorption frequencies for samples annealed for 2 h, 4 h, and 8 h (Figure 5b,c). Electric field intensity maps are reported along the side view (Figure 5b) and the top view (Figure 5c), revealing the local electric field concentration within and around the NTs. In the case of the 2 h–annealed sample, the electric field was relatively weak and broadly distributed without significant localization either inside the NTs or in the surrounding regions. A similar effect was observed for the 4 h–annealed sample, where the fields remained diffuse, although a slight enhancement appeared in the region at the bottom of the NTs. The 8 h–annealed structure exhibited a slightly more pronounced electric field confinement between adjacent NTs, particularly in the inter‐tube gaps near the top of the array (Figure 5c, bottom panel). This behavior could be explained by an increased dielectric contrast due to a higher rutile phase fraction and a thicker underlying compact oxide layer, both of which promoted more effective mode confinement. Importantly, the fields tend to zero inside the NTs for all annealing conditions. This suggests that the primary absorption mechanism at the THz resonance is governed by bulk absorption at the top part of the oxide layer along with surface and interstitial field interactions in the NT space. This conclusion agrees with the negligible effect of the NT dimensional parameters on the THz absorption peak position discussed above and can be explained by the deep subwavelength size of the NTs in this frequency range.

To further highlight the correlation between annealing time and optical properties in the THz region, the simulated peak absorption frequency (ν_peak_), the experimental absorptance, and the imaginary part of the fitted permittivity ε″ were plotted as a function of the annealing time (Figure 6). For this comparative analysis, all mentioned quantities were evaluated at the resonant frequency corresponding to each annealing duration. The peak frequency (black squares) exhibited a nonmonotonic trend with annealing time. It increased from ∼2.0 THz in the as‐anodized state to a maximum of 2.62 THz after 2 h of annealing, then redshifted progressively to 2.46 THz and 1.67 THz for 4 and 8 h, respectively. This behavior was closely linked to the material's dielectric properties, as evidenced by the evolution of the Lorentz‐fitted permittivity (Table 1). At 2 h, the real and imaginary permittivity values at ν_peak_ were ε′ = 315.3 and ε″ = 12.2, which indicates strong phonon–polariton interaction and high oscillator strength. As annealing progressed, ε′ decreased to 227.3 and 221.1, whereas ε″ reduced to 11.8 and 9.1 for 4 and 8 h, respectively. The decline in ε″ reflected a decrease in phonon damping, consistent with improved crystallinity and the dominance of the rutile phase, which supported more coherent phonon oscillations [30, 32, 34, 35]. The increase in experimental absorptance with annealing time from ∼75% to nearly 87% (red circles in Figure 6) confirmed enhanced impedance matching and resonant field confinement due to the evolving dielectric landscape. These trends strongly correlated with the thickening of the underlying compact oxide layer (from ∼1.5 to ∼3 μm) and increased rutile crystallinity revealed by structural characterization (Figure 2). Together, these data established that THz absorptance in TiO_2_ NTs is governed by an interplay of phase composition, oxide geometry, and dielectric dispersion, which can be precisely engineered through thermal treatment.

**FIGURE 6:**
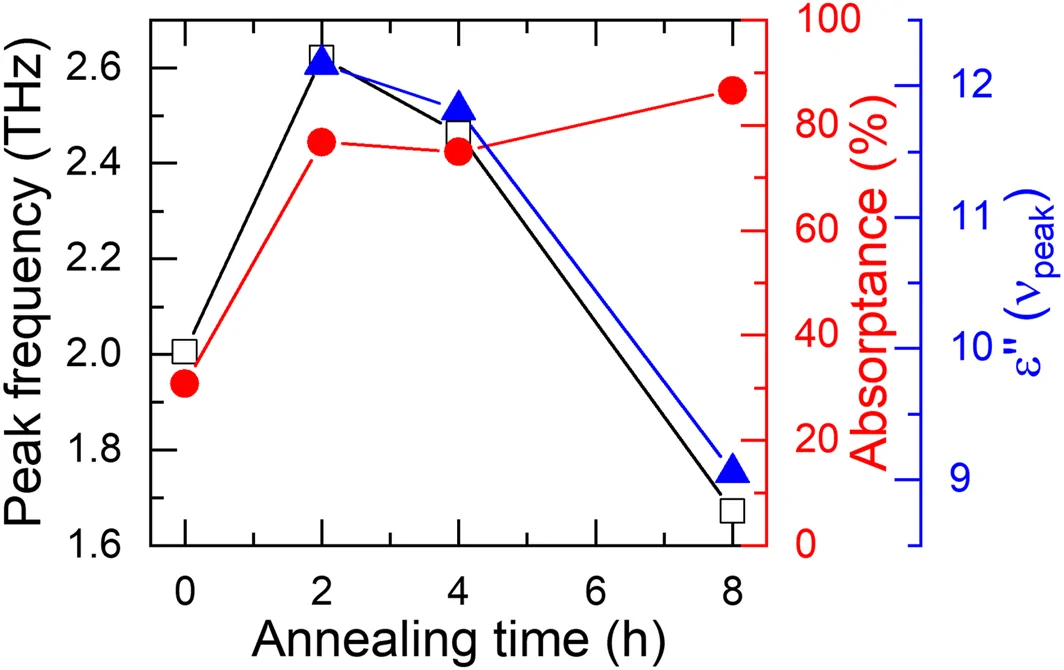
Comparison among simulated peak frequency (υ_peak_, black), absorptance (red), and imaginary part of the fitted permittivity (blue), both of which were evaluated at the peak frequency.

## 3 Conclusion

This study introduced a novel, ultrathin, and lithography‐free THz absorber based on vertically aligned spaced TiO_2_ NT arrays fabricated via electrochemical anodization followed by thermal annealing. The structure exhibited a tunable broadband absorption peak (∼1 THz wide) in the 1.4–3.6 THz range. Above 10 THz, all annealed samples exhibited consistently high absorptance, reaching approximately 80% up to 300 THz. In the visible to near‐UV range (300–1000 THz), the 2 h– and 4 h–annealed samples maintained absorptance levels above ∼80%, whereas the 8 h–annealed sample showed a moderate decrease to around 60%. By varying annealing time up to 8 hours, we demonstrated tunable THz absorption peaks shifting from 2.62 THz (2 h) to 1.67 THz (8 h) and a consistent increase in the absorptance value up to 87% (8 h). These shifts were directly linked to the anatase‐to‐rutile phase transition, which modulates the real and imaginary parts of the dielectric permittivity and reduces phonon damping. A key advantage of this experimental approach consists of a straightforward tunability of the absorption spectrum via annealing‐induced phase transformation, which modulates the dielectric function and phonon oscillator strength. Simulations incorporating Lorentz dispersion modeling and realistic structural parameters confirmed strong agreement with experimental results, capturing both spectral shifts and field confinement dynamics. The evolving compact oxide layer beneath the tubes further contributed to enhanced absorption by tuning the cavity geometry and impedance matching. Overall, the TiO_2_ NT platform offered a scalable, lithography‐free, and easily tunable approach toward subwavelength THz absorbers across an ultra‐broad spectral range. Its metrics are benchmarked against the literature of material‐based THz absorbers in Supporting Information S1 Table S2. These findings have significant implications for applications in THz beam shaping, spectroscopy, real‐time imaging, and photonic energy harvesting systems.

## 4 Experimental Methods

### 4.1 Sample Preparation

Spaced TiO_2_ NT arrays were prepared according to an already reported procedure [24, 38]. A Ti plate (1.5 × 1.5 cm^2^ size with 0.25 mm thickness cut from 99.7% purity foil, Sigma‐Aldrich, product code 267503) was ultrasonically cleaned in acetone, ethanol, and distilled water (∼15 minutes each) and dried under an N_2_ flow. The Ti plate was employed as a working electrode in a two‐electrode electrochemical cell (Pt sheet as counter electrode, 60 V voltage, 3 h, diethylene glycol electrolyte with 0.5 wt.% ammonium hydrogen difluoride and 3.6 wt.% H_2_O, 40°C). The so‐obtained spaced TiO_2_ NTs were rinsed in ethanol and distilled water, dried under an N_2_ flow, and annealed in air at 650°C for 2, 4, or 8 h with heating and cooling rates of 2°C/min.

### 4.2 Characterization

The morphology of the samples was investigated by scanning electron microscopy (SEM, Hitachi FE‐SEM 4800). The crystalline structure was investigated by X‐ray diffraction (XRD) using a high‐resolution X‐ray powder diffractometer (Empyrean, Malvern Panalytical B.V., Almelo, The Netherlands) with Co Kα radiation (λ = 0.1789 nm) operated in Bragg–Brentano geometry in a 2θ range of 20°–105° with a step size of 0.033°. The quantitative phase composition and crystallite size were determined from diffraction patterns using the Rietveld refinement method performed in MStruct software (version 2019, Charles University in Prague, Czech Republic) [39]. The irradiated volume was defined by the experimental Bragg‐Brentano geometry of the diffractometer, the effective penetration depth of the X‐ray radiation (∼3 μm), and the primary optics (Göbel mirror with a fixed divergence slit of 1/2°, mask 4 mm, and Soller slits 0.04 rad). The presented errors follow the Rietveld refinement method. The optical properties were evaluated by reflectance (*R*) measurements in an ultra‐broadband region of the electromagnetic spectrum with a combination of different experimental setups. In all cases, the absorptance (*A*) was evaluated as *A* = 1 – *R* because of the opaque nature of Ti substrates at all wavelengths without applying any scaling or normalization (see the Supporting Information for exemplary raw reflectance spectra). The absorptance in the 250–1300 nm range (UV‐vis‐NIR) was obtained from total reflectance measurements with a double‐beam spectrophotometer (Shimadzu UV‐2600) equipped with a 60 mm‐diameter integrating sphere coated with BaSO_4_ (Shimadzu ISR‐2600) using BaSO_4_ as reference. The absorptance in the 7497–400 cm^−1^ (∼1.3–25 μm, MIR) and 679–75 cm^−1^ (∼15–134 μm, FIR) ranges was obtained from specular reflectance measurements with a vacuum Fourier‐transform infrared (FTIR) spectrometer (Vertex 70v, Bruker) using Au as a reference. The absorptance in the THz range was obtained using two different systems in reflection mode. First, a TOPTICA TeraScan 1550 CW photomixer was employed along with a reflection THz head for measurements in the 0.1 to 3.1 THz range. The reflection head contained four off‐axis parabolic mirrors (each 1″ in diameter) arranged to direct, focus, and collect the THz beam. The two mirrors near the antennas had a 2″ focal length, whereas the two central mirrors had a 4″ focal length. Together, they formed a compact optical path that focused the THz radiation on the sample and re‐focused the specular reflected signal onto the receiver antenna. The mirrors were slightly tilted, providing an 8° incidence angle of the THz beam on the sample surface. At 1 THz, the focal spot size was approximately 1 mm in diameter. A lock‐in integration time of 300 ms was used, yielding a dynamic range of ~110 dB at 100 GHz and ~40 dB at 3 THz. Second, a Menlo Tera K15 Mark II THz‐TDS system was employed in the 0.1–2 THz range. Broadband THz pulses generated by the THz emitter (Tera 15 SL25) were focused onto the sample using a TPX35 lens. The reflected signal retraced the same path and was directed to the THz detector (Tera 15 DP25) via a silicon beamsplitter. Time‐domain scans were acquired over a 0–40 ps window. The beam spot diameter on the sample was approximately 2 mm at 1 THz [40]. Under these settings and in unpurged conditions, the system had a peak dynamic range of ~65 dB and provided a usable bandwidth from 0.1 to 2 THz.

### 4.3 Numerical Modeling

The electromagnetic response was numerically investigated using the frequency‐domain solver of CST Studio 2025. A single NT unit cell was modeled using Unit cell boundary conditions along the *x*‐ and *y*‐directions to capture the collective lattice interaction. Along the *z*‐direction (normal to the substrate), open boundaries with software‐defined Floquet ports were applied, supporting 18 Floquet modes to ensure accurate simulation of higher‐order diffraction and field propagation. The tangential electric field component at the lower *z*‐boundary was set to zero (*E*_*t*_ = 0) to enforce convergence and accurately model radiation behavior under oblique and diffractive incidence.

The geometrical parameters used in the simulation were consistent with the fabricated samples: square array pitch *p* = 480 nm, inner diameter *d*_*in*_ = 215 nm, outer diameter *d*_*out*_ = 280 nm, and NT height *h* = 3.8 μm, which remained constant across all annealing durations. The substrate was modeled as a perfect electric conductor (PEC) of thickness 0.27 μm to emulate the titanium foil's highly reflective response in the THz regime. A compact rutile layer beneath the NTs of 1.6, 2.0, and 3.0 μm thickness for the 2, 4, and 8 h annealed samples, respectively, was also included.

The frequency‐dependent dielectric response of TiO_2_ (both NTs and compact rutile layer) was modeled using a single Lorentz oscillator dispersion model by using Equation (2) as given below, which captures the material's frequency‐dependent complex permittivity due to phonon–polariton interactions [17] in the THz range:

(2) $$\varepsilon \left(\omega \right)=\varepsilon_{\infty }+\frac{\left(\varepsilon_{s}-\varepsilon_{\infty }\right)\omega_{0}^{2}}{\omega_{0}^{2}+i\omega \delta -\omega^{2}}$$

Here, $\varepsilon_{s}$ and $\varepsilon_{\infty }$ denote the static and high‐frequency permittivities, respectively, $\omega_{0}$ is the phonon resonance angular frequency (in rad s^−1^), and δ is the damping constant (in s^−1^). The values of the Lorentz oscillator parameters reported in ref. [17] were used as starting values for the fit of the experimental absorption peak in the THz region for each sample, which is motivated by the prevalence of the rutile phase in all annealed NTs.

## Author Contributions

**Anjali Chaudhary:** investigation, writing – original draft, methodology, resources, software, visualization, writing – review and editing, formal analysis, data curation. **Morteza Afshar:** investigation, methodology, formal analysis. **Emil J. Y. Magaway:** investigation, software, methodology. **Sunil Kumar:** supervision. **Ravendra Kumar Varshney:** supervision. **Jiří Čapek:** investigation, methodology, resources, validation. **Ivan Richter:** funding acquisition, supervision. **Štěpán Kment:** supervision, funding acquisition. **Miguel Navarro‐Cía:** software, methodology, writing – review and editing, project administration, resources, supervision, funding acquisition, visualization, validation, conceptualization, formal analysis. **Luca Mascaretti:** data curation, writing – review and editing, investigation, project administration, methodology, resources, visualization, validation, writing – original draft, conceptualization, funding acquisition.

## Conflicts of Interest

The authors declare no conflicts of interest.

## Supporting information


Supporting Information S1

## Data Availability

The data that support the findings of this study are available from the corresponding author upon reasonable request.
