# Supplementary material for: Ultra‐Broadband Optical Characterization of TiO2 Nanotube Arrays Revealing Annealing‐Tunable THz Absorption
Source: Nanophotonics. 2026 Jul 29;15(15):e70231. doi: 10.1002/nap2.70231 (PMC13642492; doi:10.1002/nap2.70231)
Supplement: Supplementary file 1 [file NAP2-15-e70231-s001.pdf]

Supporting Information

Ultra-broadband optical characterization of TiO_2 nanotube arrays revealing annealing-tunable THz absorption

Anjali Chaudhary, Morteza Afshar, Emil J. Y. Magaway, Sunil Kumar, Ravendra Kumar Varshney, Jiří Čapek, Ivan Richter, Štěpán Kment, Miguel Navarro-Cía, and Luca Mascaretti**

Additional characterization results

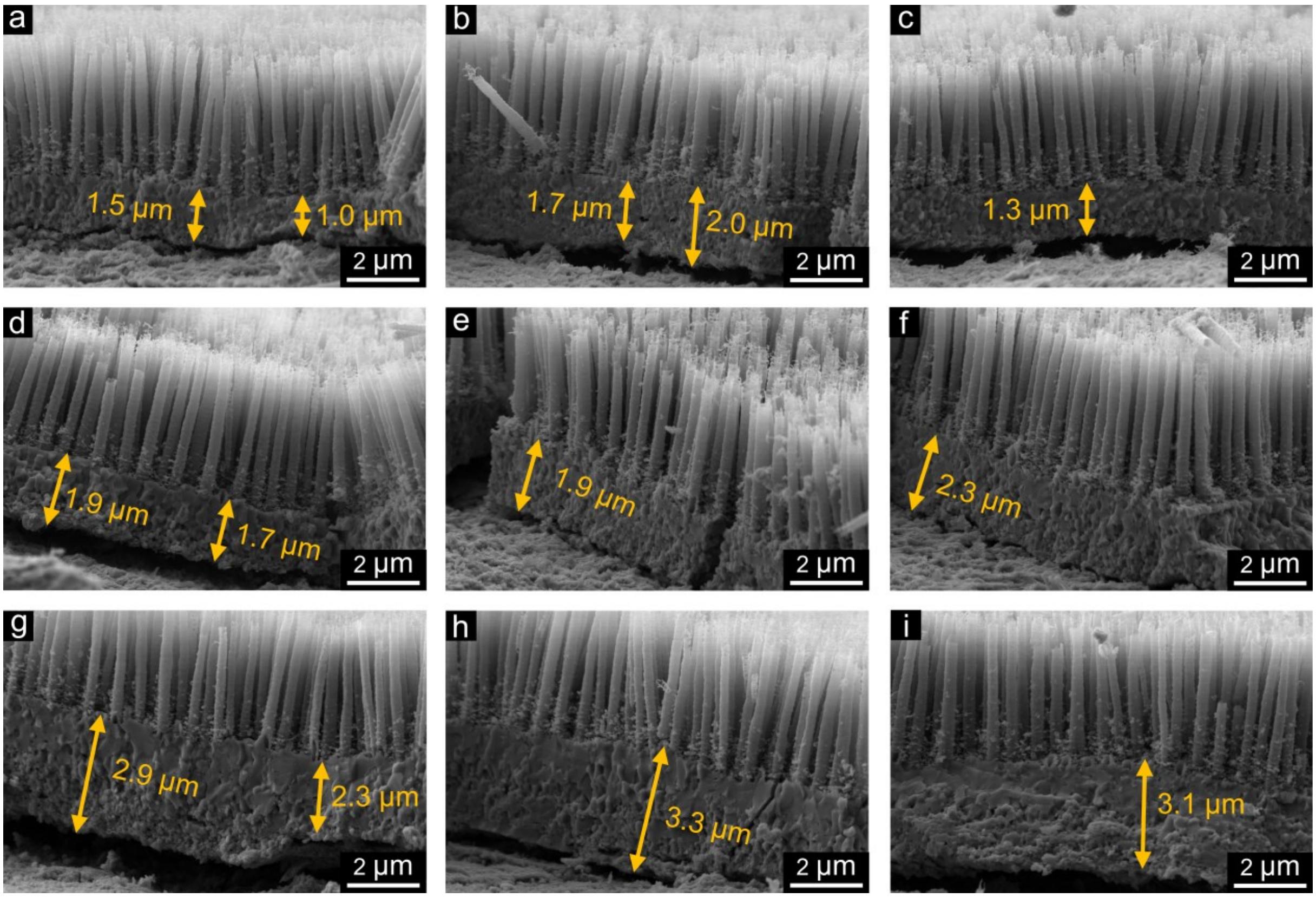

Figure S1. Cross-sectional images for the spaced TiO_2 NTs annealed in air at 650 °C for (a–c) 2 h, (d–f) 4 h and (g–i) 8 h.

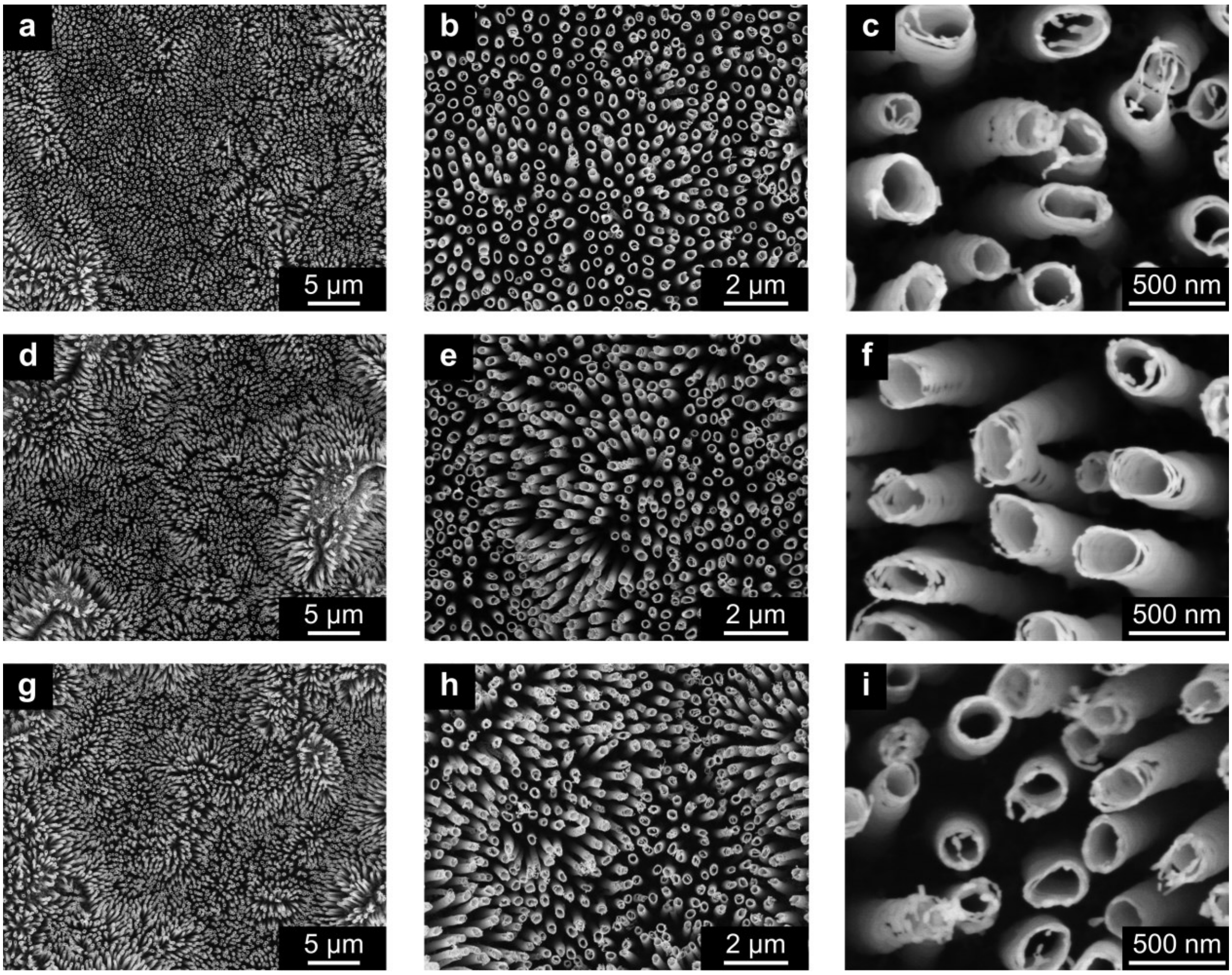

Figure S2: Top-view SEM images of spaced TiO_2 nanotubes (NTs) annealed in air at 650 °C for (a–c) 2 h, (d–f) 4 h and (g–i) 8 h.

Additional cross-sectional and top-view SEM images of the spaced TiO_2 NT arrays annealed for different durations are provided in **Figures S1** and **S2**. The images show the increase of the thickness of the compact rutile layer beneath the NTs with the annealing time, i.e., ~ 1.5, ~ 2 and ~ 3 µm for 2, 4 and 8 h, respectively, with some non-uniformities mainly ascribed to the roughness of the Ti substrates. On the other hand, the top-view images confirm that the overall NT morphology, including the inter-tube spacing and ordering, remains largely unchanged with increasing annealing time, indicating that the thermal treatment primarily affects the crystalline phase and underlying oxide layer rather than the surface geometry of the NT array.

Table S1. XRD analysis of spaced NTs with corresponding Miller indices for the anatase (A), rutile (R) TiO_2 phases and metallic titanium (Ti). The intensity of the diffraction peaks was normalized to the main rutile (110) peak.

	**2 h**		**4 h**		**8 h**	
**Assignment**	**2θ (°)**	**I**	**2θ (°)**	**I**	**2θ (°)**	**I**
A(101)	29.749	0.51	29.582	0.39	29.649	0.26
R(110)	32.256	1.00	32.055	1.00	32.122	1.00
R(101)	42.383	0.25	42.216	0.20	42.349	0.21
A(103)	43.452	0.04	43.218	0.03	43.419	0.02
A(004)	44.455	0.14	44.321	0.11	44.355	0.05
R(200)	46.160	0.05	45.993	0.05	46.093	0.05
Ti(101)	47.229	0.17	47.062	0.17	47.229	0.06
R(111)	48.499	0.12	48.399	0.10	48.466	0.10
R(210)	51.841	0.06	51.708	0.05	51.841	0.04
A(200)	56.721	0.08	56.554	0.05	56.621	0.04
Ti(102)	62.537	0.18	62.403	0.22	62.503	0.12
A(105)	63.840	0.07	63.773	0.06	63.706	0.03
R(211)	64.275	0.25	64.141	0.23	64.241	0.23
A(211)	65.244	0.04	65.077	0.03	65.144	0.02
R(220)	67.149	0.10	67.015	0.11	67.082	0.11
R(002)	74.636	0.07	74.469	0.05	74.569	0.05
R(310)	76.307	0.04	76.106	0.03	76.206	0.03

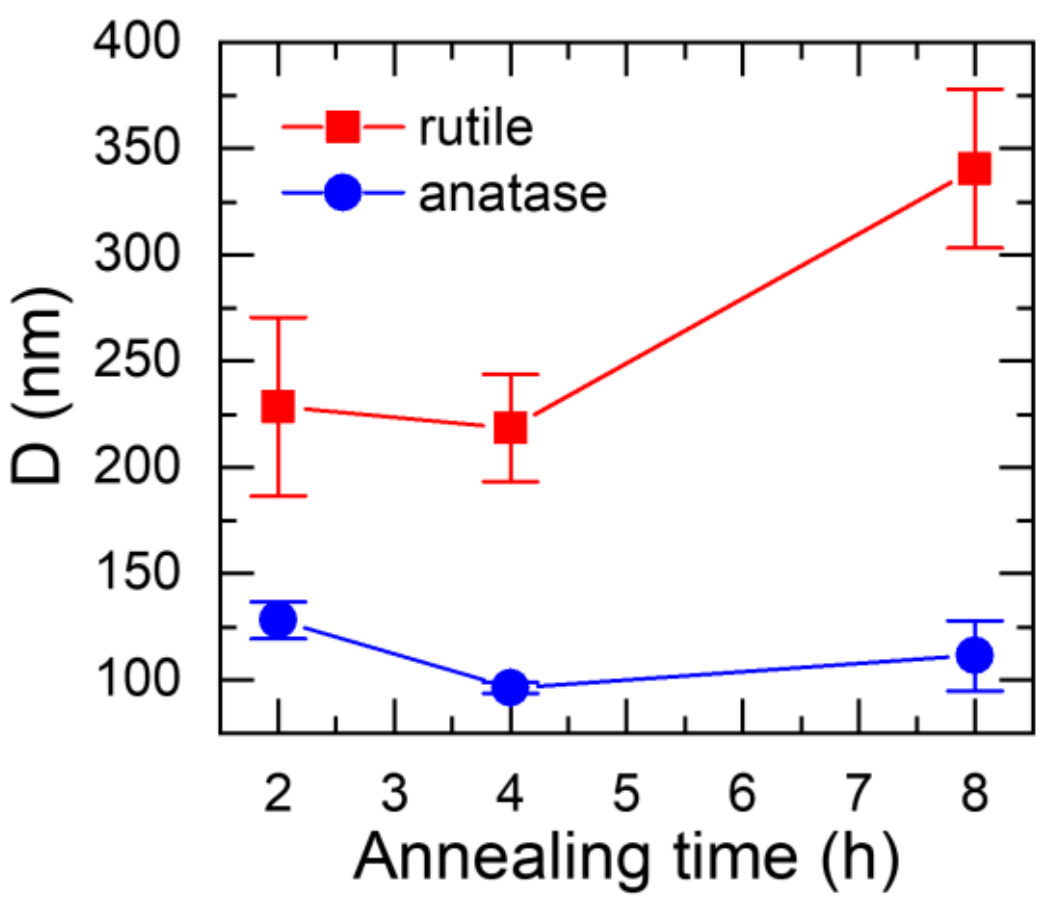

Figure S3. Average crystallite size obtained by Rietveld refinement method for the spaced TiO_2 NTs vs. annealing time from the (110) and (101) diffraction peaks of rutile (red) and anatase (blue), respectively.

Figure S3 shows the evolution of the average crystallite size of the TiO_2 NTs as a function of annealing time estimated using the Rietveld refinement method in MStruct software.[1] The crystallite size of the rutile phase increases with annealing time, indicating enhanced crystal growth and improved crystallinity during thermal treatment. In contrast, the crystallite size of the anatase phase remains nearly constant. This trend is consistent with the progressive anatase-to-rutile phase transformation and the increasing dominance of the rutile phase at longer annealing durations.

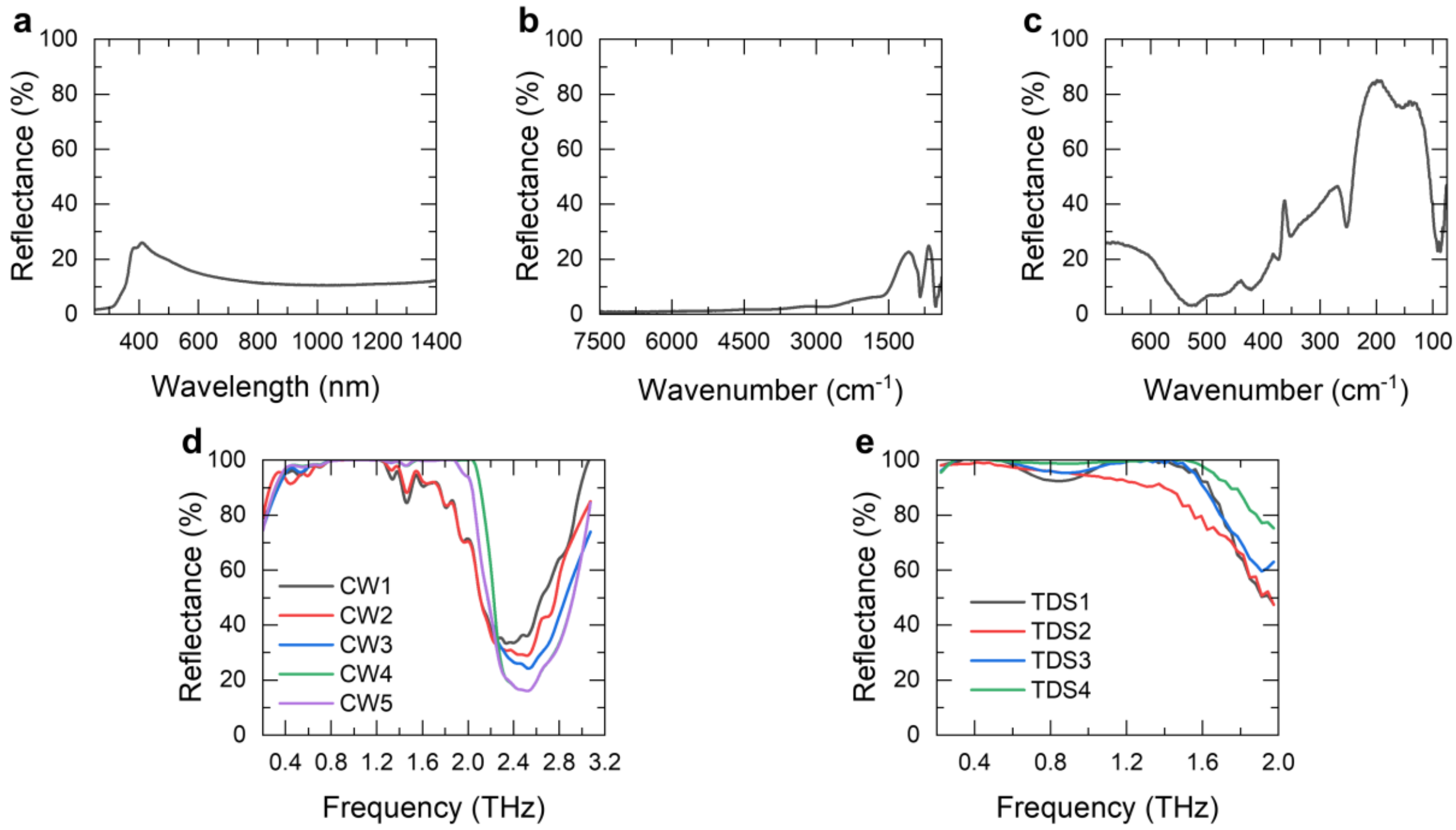

Figure S4. Raw reflectance data for the 2 h-annealed NTs as measured by the spectrophotometer in the UV-vis-NIR range (a), by the FTIR spectrometer in the medium-IR range (b) and far-IR range (c), by the CW system (d), and by the TDS system (e) in the THz range.

Measurements with the spectrophotometer and with the FTIR spectrometer consisted of single-point acquisitions, while those with the CW and TDS systems consisted of multiple acquisitions for data averaging. Furthermore, total reflectance was measured in the UV-vis-NIR range (a) since the spectrophotometer was equipped with an integrating sphere, unlike all other cases, which consisted in specular reflectance measurements. This explains the mismatch between the reflectance data in (a) and (b) at ~ 1300 nm/7500 cm^{-1}.

Additional simulation results

The influence of the compact TiO_2 oxide layer thickness on the THz absorptance was investigated for both TE and TM polarizations **(Figure S5)**. For all annealing durations, variations in oxide layer thickness led to a noticeable shift of the absorption resonance, confirming that the THz response is highly sensitive to the underlying oxide thickness. Similar spectral behavior is observed for both TE and TM polarizations, indicating that the absorption mechanism is predominantly governed by the intrinsic dielectric response of the TiO_2 layer rather than polarization-dependent geometrical effects of the NT array.

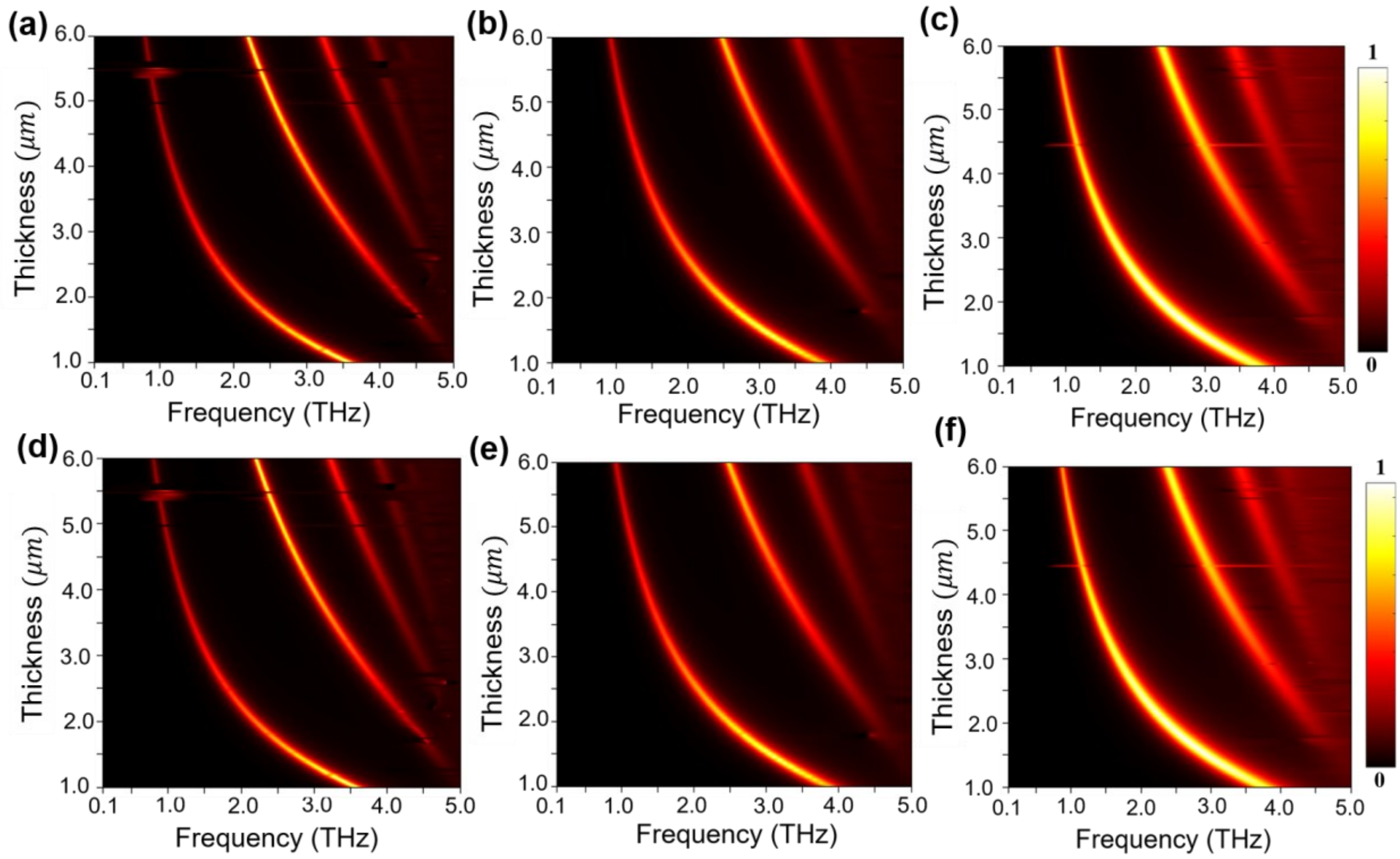

Figure S5. Simulated normalized absorptance spectra showing the effect of compact TiO_2 layer thickness variation under TE polarization: (a) 2 h, (b) 4 h, and (c) 8 h annealed samples; and under TM polarization**:** (d) 2 h, (e) 4 h, and (f) 8 h annealed samples.

The comparison between experimentally measured and simulated absorptance spectra shown in the main text **(Figure 5a)** reveals a noticeable discrepancy in the resonance linewidth, where the experimental absorption peak appears significantly broader than the simulated one. This difference indicates that the experimentally investigated samples cannot be described by a perfectly uniform structure. In the simulations, an idealized geometry with a uniform compact TiO_2 oxide layer thickness is assumed, resulting in a relatively narrow resonance determined by a single well-defined phonon–polariton condition. In contrast, during the anodization and thermal annealing processes, the growth of the compact oxide layer beneath the NTs is inherently non-uniform across the sample surface. As a consequence, different regions of the sample possess slightly different oxide thicknesses. Since the THz absorption resonance strongly depends on the oxide layer thickness, each local thickness supports a

resonance at a slightly different frequency (**Figure S5**). The experimentally measured spectrum therefore represents the spatial averaging of multiple resonances, leading to an overall broadened absorption peak.

This interpretation is further supported by **Figure S6**, which presents an indirect estimation of the variations of the compact rutile layer thickness for different annealing durations retrieved from absorption spectra in the THz region. The central square markers denote the compact layer thickness corresponding to the resonance frequency (ν_{peak}) obtained from simulations matched with the experimental data (**Figure 5a** in the main text), while the error bars represent the thickness range required to reproduce the experimentally observed absorption linewidth. The increasing deviation indicates non-uniform oxide layer growth during annealing, resulting in spatially distributed resonance conditions and, consequently, broadening of the experimental absorption peaks. These results provide evidence of the compact layer non-uniformity and agree with the experimental observations of thickness non-uniformities presented in **Figure S1**.

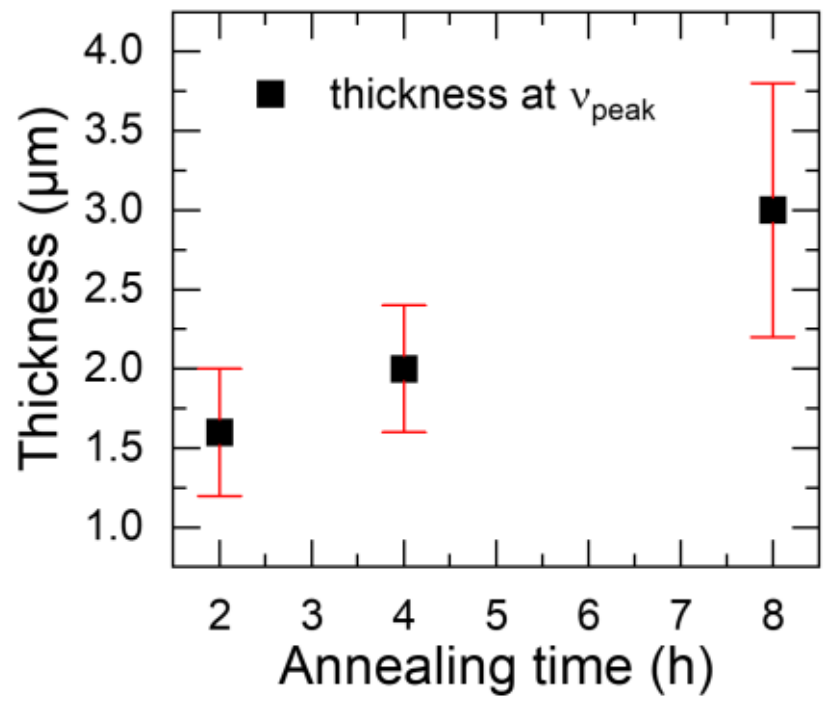

Figure S6. Deviation of the compact TiO_2 layer thickness for different annealing durations retrieved by the analysis of the main absorption peak in the THz region.

To estimate oxide layer thickness variation, a fixed reference absorption level (approximately 50% absorption) was selected independently for each annealing condition. The lower- and upper-frequency limits were determined from the experimental spectra as the frequencies at which the absorption curve intersects this reference level. Simulated spectra were then used to identify the oxide thickness values that reproduce these frequency positions while keeping the fitted dielectric response unchanged. This approach provides an estimate of the effective oxide thickness range contributing to the experimentally observed spectral broadening.

For the 2 h annealed sample, the nominal resonance occurs at 2.26 THz with an oxide thickness of ~ 1.6 µm. At the chosen reference absorption level, the lower-frequency edge appears at 2.17 THz,

corresponding to a simulated oxide thickness of approximately 2.0 µm, while the upper-frequency edge occurs at 3.17 THz, corresponding to approximately 1.25 µm. This indicates an effective oxide thickness range of 1.25–2.0 µm, corresponding to deviations of roughly ±0.4 µm relative to the nominal thickness.

For the 4 h annealed sample, the resonance shifts to 2.46 THz with a nominal oxide thickness of ~ 2.0 µm. The lower-frequency edge at the reference absorption level is 2.13 THz, matched by simulations with ~2.4 µm, while the upper-frequency edge at 3.02 THz corresponds to approximately 1.6 µm. The inferred oxide thickness range is therefore 1.6-2.4 µm, corresponding to a deviation of approximately ±0.4 µm.

For the 8 h annealed sample, the nominal resonance occurs at 1.67 THz with an oxide thickness of ~ 3.0 µm. The lower-frequency edge at 1.30 THz corresponds to an oxide thickness of approximately 3.8 µm, whereas the upper-frequency edge at 2.32 THz corresponds to approximately 2.2 µm. The resulting oxide thickness range extends from 2.2 to 3.8 µm, indicating a larger deviation of approximately ±0.8 µm around the nominal thickness. Overall, the analysis shows that thinner oxide regions contribute to higher-frequency responses, whereas thicker regions contribute to lower-frequency responses. The superposition of contributions from regions with different oxide thicknesses leads to the experimentally observed spectral broadening.

The influence of NT geometrical parameters on the THz absorptance was further investigated through simulations by varying the NT diameter (**Figure S7**), height (**Figure S8**), and pitch (**Figure S9**) under both TE and TM polarizations. For all annealing durations, the simulated spectra exhibit nearly identical responses with only negligible shifts in the resonance frequency and minimal variation in absorptance. These results indicate that the THz absorption is largely insensitive to the geometrical parameters of the NT array. Instead, the resonance is primarily governed by the intrinsic dielectric properties of TiO_2 and the thickness of the compact oxide layer rather than by the NT geometry or lattice periodicity.

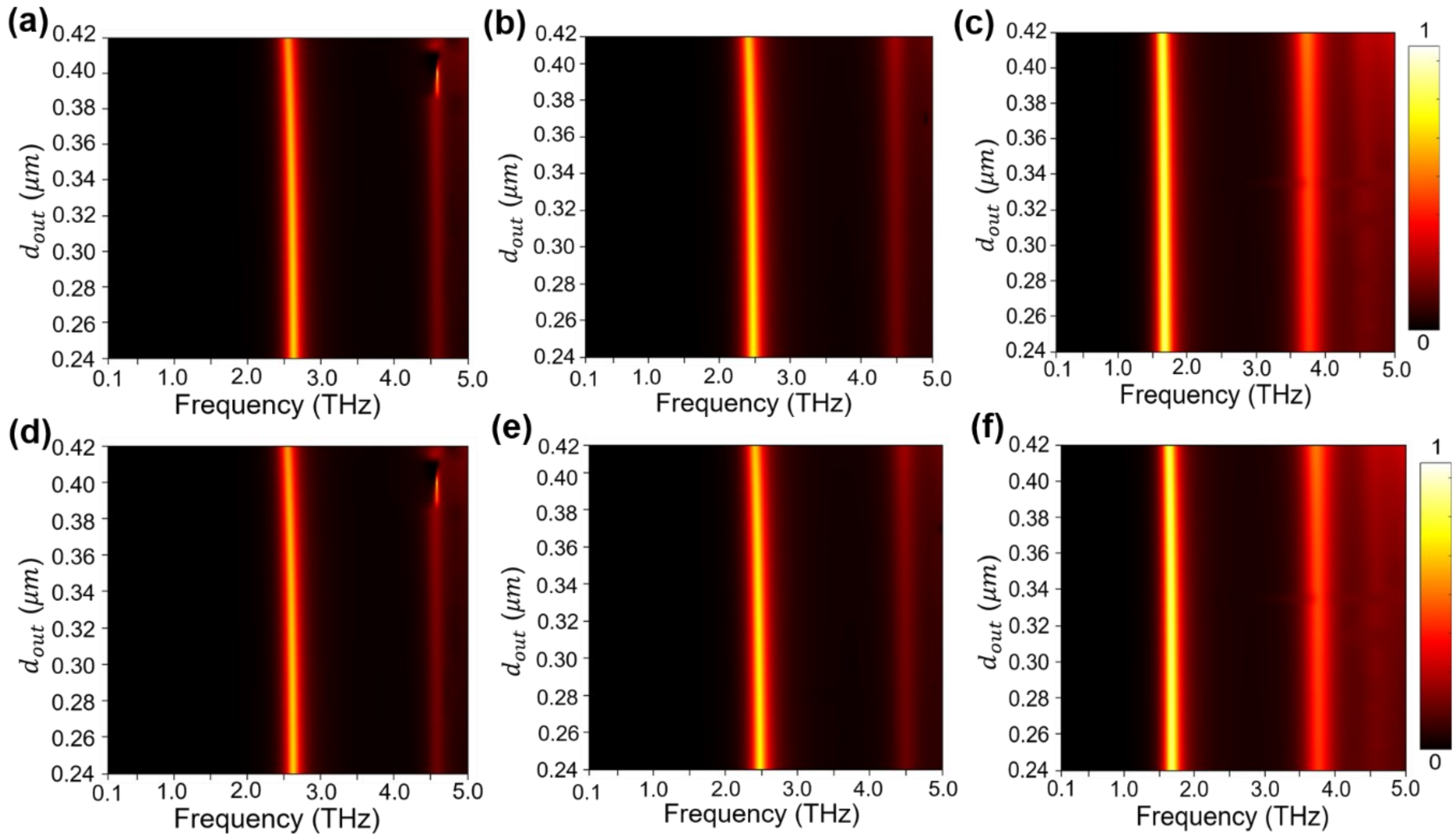

Figure S7. Simulated normalized absorptance spectra showing the effect of NT diameter variation for TiO_2 NT samples under TE polarization: (a) 2 h, (b) 4 h, and (c) 8 h annealed samples; and under TM polarization: (d) 2 h, (e) 4 h, and (f) 8 h annealed samples.

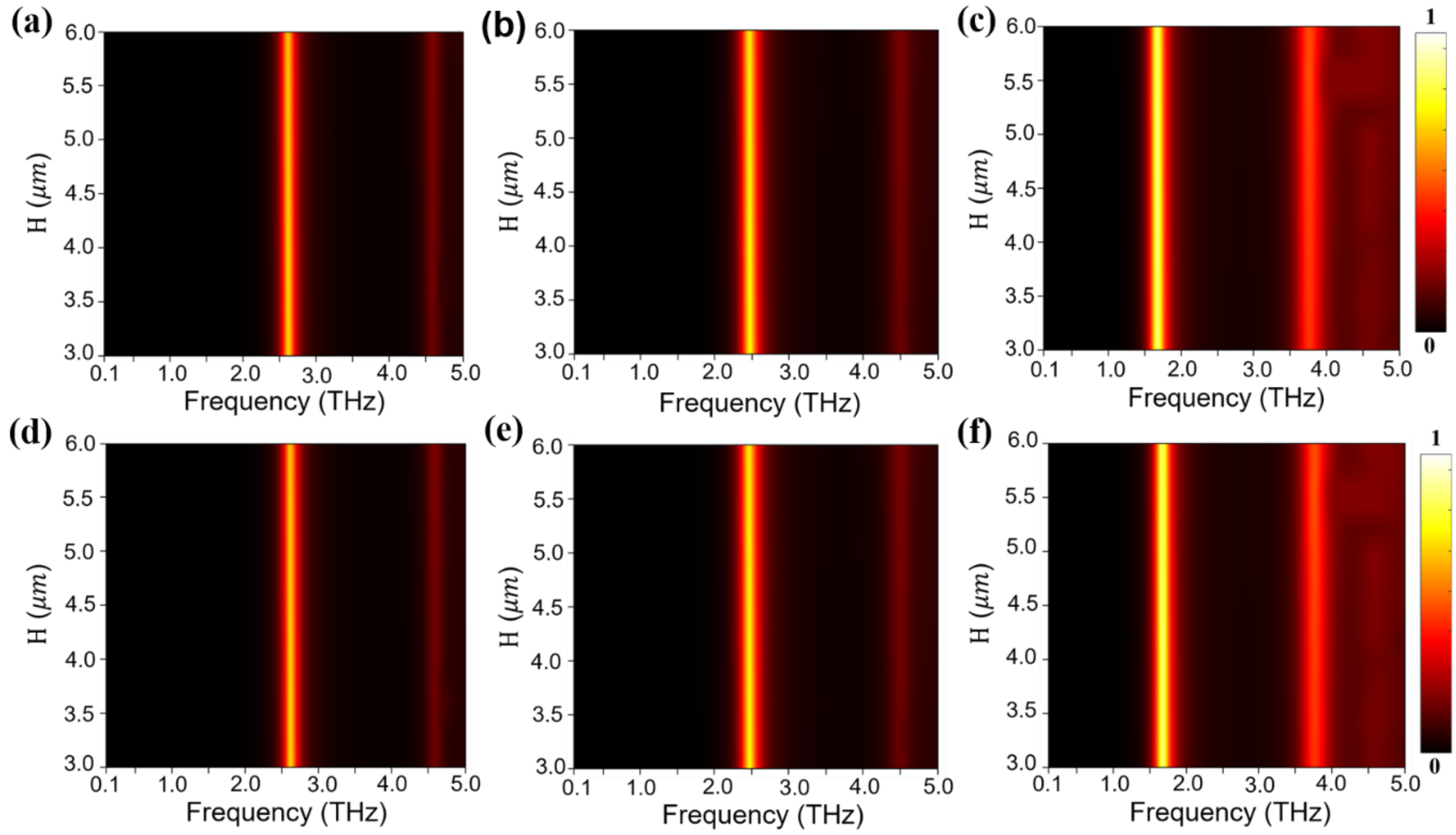

Figure S8. Simulated normalized absorptance spectra showing the effect of NT height variation for TiO_2 NT samples under TE polarization: (a) 2 h, (b) 4 h, and (c) 8 h annealed samples; and under TM polarization**:** (d) 2 h, (e) 4 h, and (f) 8 h annealed samples.

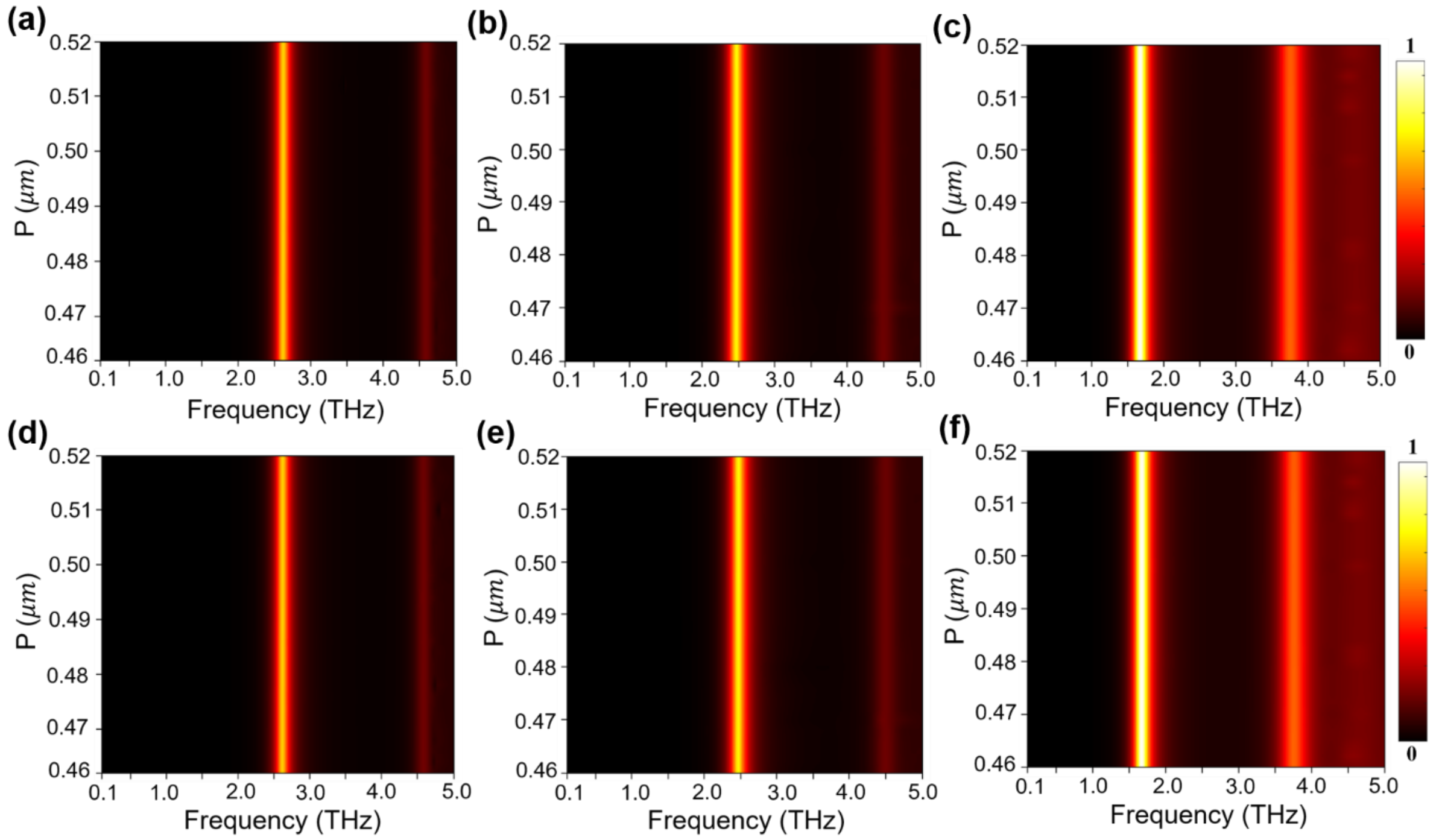

Figure S9. Simulated normalized absorptance spectra showing the effect of pitch for TiO_2 NT samples under TE polarization: (a) 2 h, (b) 4 h, and (c) 8 h annealed samples; and under TM polarization**:** (d) 2 h, (e) 4 h, and (f) 8 h annealed samples.

In the terahertz regime (0.1–5 THz)**,** the electromagnetic wavelength (λ ~ 60–3000 μm) is orders of magnitude larger than the NT dimensions (hundreds of nm to a few μm). Under this deep subwavelength condition, the NT array behaves as an effective homogeneous medium, and the absorption response is primarily governed by the intrinsic phonon-polariton dielectric properties of TiO_2 and the thickness of the compact oxide layer. Consequently, variations in NT diameter, height, or pitch produce only negligible changes in the THz absorption characteristics.

Across all investigated samples (see **Figure 4** in the main text), the broadband absorption behavior is governed by the interplay between the intrinsic dielectric response of TiO_2 and impedance matching conditions at the air-oxide interface. Although TiO_2 exhibits a large real permittivity (ε′) in the terahertz regime, strong absorption requires not only a high refractive index but also sufficient dielectric loss, represented by the imaginary permittivity component (ε″).

In the low-frequency THz region, the observed absorption peaks originate from phonon-polariton excitation associated with lattice vibrations in TiO_2 [2–8]. Near these resonances, ε″ becomes sufficiently large, enabling efficient electromagnetic energy dissipation and improved impedance matching with free space, which results in enhanced absorptance.

However, in the intermediate frequency range (~5–10 THz), all samples exhibited a noticeable reduction in absorption. In this spectral region, the excitation frequency moves away from the dominant phonon

resonance, leading to a decrease in dielectric damping and consequently lower ε″ values. Despite maintaining a relatively large ε′, the reduced material loss prevents efficient energy dissipation within the oxide layer. As a result, the TiO_2 layer behaves effectively as a high-index dielectric film supported by a metallic Ti substrate, where reflection dominates over absorption. Under these conditions, impedance matching deteriorates and the absorptance decreases.

Furthermore, within this frequency window the NT dimensions remain deeply subwavelength compared to the incident wavelength, preventing the emergence of geometry-induced resonant or scattering mechanisms that could compensate for the reduced intrinsic material loss. Consequently, the absorption minimum observed between 5 and 10 THz represents an intrinsic material-driven transition region rather than a geometrical effect. At higher frequencies (>10 THz), absorption increases again due to the activation of higher-order infrared-active lattice vibrations and multi-phonon processes in TiO_2, which enhance dielectric losses and restore efficient electromagnetic energy dissipation.

The angular dependence of the THz absorptance was investigated through simulations by varying the incident angle (θ) under both TE and TM polarizations **(Figure S10)**. For TE-polarized incidence, the resonance frequency remains nearly invariant with increasing incident angle, while the absorption intensity gradually decreases at higher θ. This occurs because the electric field component remains predominantly parallel to the surface, preserving the excitation condition of the phonon–polariton resonance, whereas the effective coupling strength reduces due to increased reflection and impedance mismatch at oblique incidence.

In contrast, under TM polarization, the resonance peak exhibits a slight shift toward higher frequencies as θ increases, accompanied by a reduction in absorptance. This behavior arises from the presence of a non-zero electric field component normal to the surface at oblique incidence, which modifies the effective optical path length and electromagnetic boundary conditions inside the compact TiO_2 oxide layer. Consequently, the resonance condition is slightly altered, leading to a small blueshift of the absorption peak.

Despite these polarization-dependent angular effects, the resonance remains well preserved over a wide angular range, confirming that the THz absorption mechanism is dominated by intrinsic phonon-polariton excitation in the TiO_2 oxide layer rather than geometrical resonances of the nanotube array.

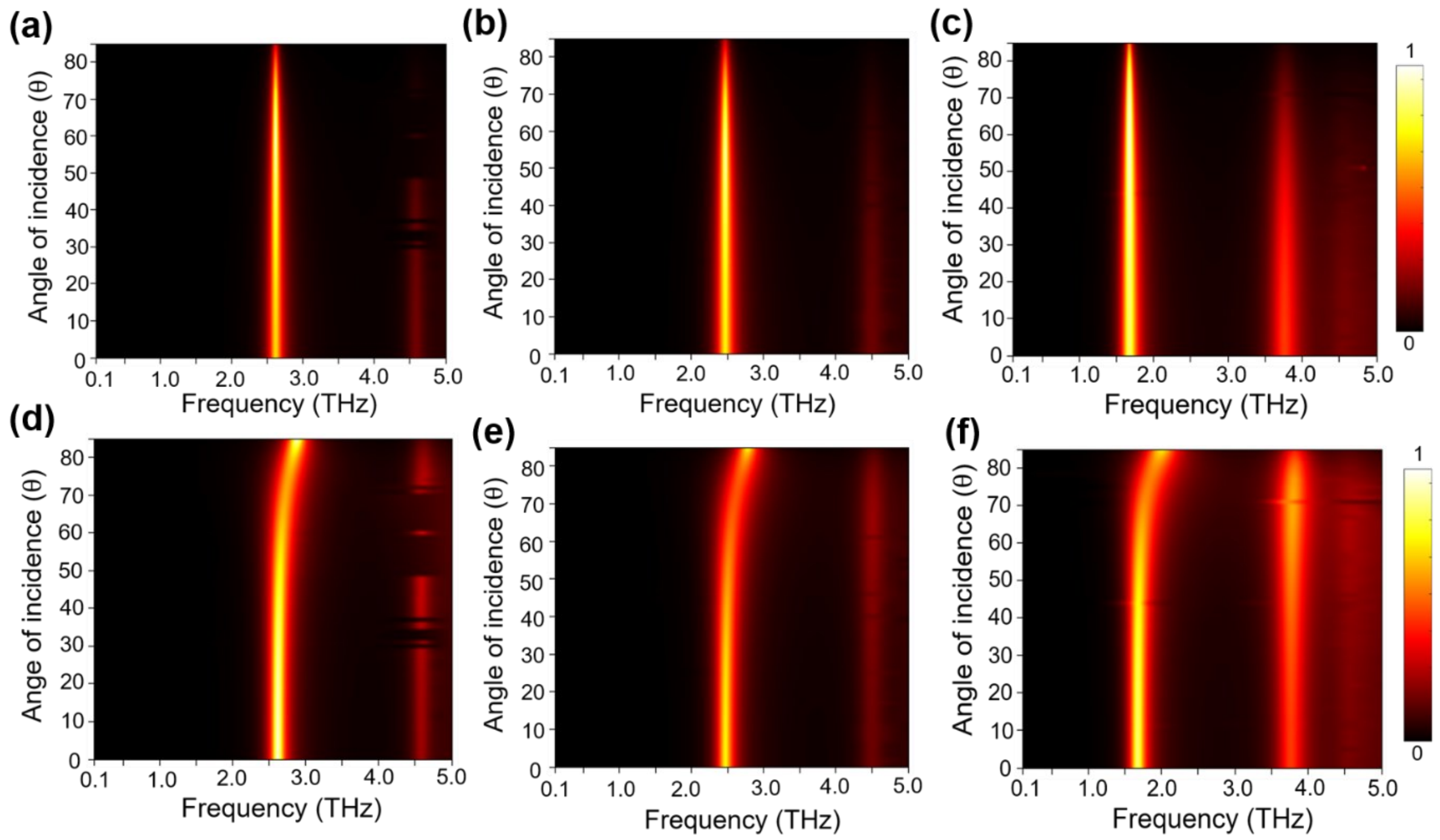

Figure S10. Simulated normalized absorptance spectra as a function of incidence angle (θ) for TiO_2 NT samples under TE polarization: (a) 2 h, (b) 4 h, and (c) 8 h annealed samples; and under TM polarization: (d) 2 h, (e) 4 h, and (f) 8 h annealed samples.

Benchmarking with current literature

For completeness, a state-of-the-art comparison of representative TiO_2 and other material-based THz absorbers is provided in **Table S2** of the Supplementary Information, placing the present work in the context of the existing literature.

Table S2. State-of-the-art comparison of representative TiO_2 and other material-based THz absorbers.

Ref.	Material/System	Freq. Range (THz)	Absorption	Tunability	Absorption Mechanism
[9]	3D titanium nitride (TiN) nanopillars	130–1000	~94%	geometry tuning	gap plasmon resonance, guided resonance, Mie resonance, low-Q resonator
[10]	vertically aligned carbon nanotube arrays (VACNT)	0.1–2.5	>96.8%	✗	intrinsic broadband CNT absorption
[11]	nanoporous titanium oxynitride nanotubes (TiO_xN_y)	200–1000	~100%	limited (porosity engineering)	plasmonic TiO_xN_y + nanoporous scattering
[12]	carbon-coated Si moth-eye	1–1200	~98%	✗	gradient-index impedance matching
[13]	black nickel coating on Cu substrate	3–1500	~95%	✗	hierarchical micro/nanostructure and multiple scattering
**This Work**	**spaced TiO_2 nanotube arrays**	**0.1–1000**	**75-87% tunable THz resonance with high broadband absorptance across the mid-IR (3-100 THz)**	**resonance tuned from 2.62 to 1.67 THz**	**annealing-induced dielectric evolution, anatase to rutile transformation, compact oxide layer formation, impedance matching, and THz resonance tuning**

References

[1] Z. Matěj, A. Kadlecová, M. Janeček, L. Matějová, M. Dopita, R. Kužel, *Powder Diffr.* **2014**, *29*, S35.

[2] S. Schöche, T. Hofmann, R. Korlacki, T. E. Tiwald, M. Schubert, *J. Appl. Phys.* **2013**, *113*, 164102.

[3] K. Kanehara, T. Hoshina, H. Takeda, T. Tsurumi, *J. Ceram. Soc. Jpn.* **2015**, *123*, 303.

[4] B. Wehinger, A. Bosak, P. T. Jochym, *Phys. Rev. B* **2016**, *93*, 014303.

[5] F. A. Grant, *Rev. Mod. Phys.* **1959**, *31*, 646.

[6] R. J. Gonzalez, R. Zallen, H. Berger, *Phys. Rev. B* **1997**, *55*, 7014.

[7] R. Sikora, *J. Phys. Chem. Solids* **2005**, *66*, 1069.

[8] P. D. Mitev, K. Hermansson, B. Montanari, K. Refson, *Phys. Rev. B* **2010**, *81*, 134303.

[9] M. Chirumamilla, A. Chirumamilla, Y. Yang, A. S. Roberts, P. K. Kristensen, K. Chaudhuri, A. Boltasseva, D. S. Sutherland, S. I. Bozhevolnyi, K. Pedersen, *Adv. Opt. Mater.* **2017**, *5*, 1700552.

[10] D. Xiao, Q. Wang, Z. Wang, Y. Zhang, J. Wu, K. Fan, L. Sun, M. Zhu, Z. K. Ng, E. H. T. Teo, F. Hu, *Adv. Opt. Mater.* **2022**, *10*, 2201363.

[11] M. Afshar, A. Schirato, L. Mascaretti, S. M. H. Hejazi, M. Shahrezaei, G. Della Valle, P. Fornasiero, Š. Kment, A. Alabastri, A. Naldoni, *ACS Photonics* **2023**, *10*, 3291.

[12] Y. Hakamada, M. Cojocari, M. Matoba, S. Kawano, H. Sakurai, K. Konishi, D. Pashnev, S. Revanth Ayyagari, V. Janonis, A. Urbanowicz, S. Tumėnas, J. Jorudas, I. Kašalynas, J. Pennanen, A. Deborah Amos, A. Saushin, G. Fedorov, Y. Svirko, P. Kuzhir, *Adv. Opt. Mater.* **2025**, *13*, 2500948.

[13] A. Menon, H. Maltanava, N. Belko, M. Cojocari, M. Gorbun, A. Saushin, K. Tamarov, J. T. T. Leskinen, M. Selenius, S. Suvanto, D. Semenov, S. Malykhin, V.-P. Lehto, G. Fedorov, P. Kuzhir, *ACS Omega* **2026**, *11*, 10723.
